# Phytochemical Analysis and Phytometabolomic Profiling of *Ficus lindsayana* Leaf Extract with Evaluation of Antioxidant, Anti-Inflammatory, Cyto- and Genotoxic Activities

**DOI:** 10.3390/ijms26199374

**Published:** 2025-09-25

**Authors:** Arisa Imsumran, Woorawee Inthachat, Piya Temviriyanukul, Jirarat Karinchai, Tanongsak Laowanitwattana, Pensiri Buacheen, Ararat Jaiaree, Uthaiwan Suttisansanee, Ariyaphong Wongnoppavich, Pornsiri Pitchakarn

**Affiliations:** 1 Department of Biochemistry, Faculty of Medicine, Chiang Mai University, Muang Chiang Mai, Chiang Mai 50200, Thailand; arisa.bonness@cmu.ac.th (A.I.); jirarat.karin@gmail.com (J.K.); tanongsak.l@cmu.ac.th (T.L.); pensiri.bua@cmu.ac.th (P.B.); ararat.ja@gmail.com (A.J.); ariyaphong.w@cmu.ac.th (A.W.); 2 Institute of Nutrition, Mahidol University, Salaya, Nakhon Pathom 73170, Thailand; woorawee.int@mahidol.ac.th (W.I.); piya.tem@mahidol.ac.th (P.T.); uthaiwan.sut@mahidol.ac.th (U.S.)

**Keywords:** fig, network pharmacology, oxidative stress, phytochemicals, proinflammatory mediators, toxicological assessment, wing spot test

## Abstract

*Ficus lindsayana* is recognized for its medicinal properties, with previous studies highlighting the antioxidant and anti-inflammatory effects of its latex (FLtA) and root (FRE) extracts. Harvesting these plant parts, however, raises ecological concerns. This study evaluates the phytochemical profiles, safety, and biological activities of *F. lindsayana* leaf (FL) extracts as more sustainable alternatives. Leaves were extracted using hot water (FLA) and 80% ethanol (FLE), yielding 32.9% and 11.4%, respectively. Metabolomic and targeted HPLC-MS/MS analyses revealed distinct phytochemical compositions. FLE was enriched in flavonoid aglycones and lipophilic compounds, while FLA contained higher levels of polar phenolics. FLA showed greater total phenolic and flavonoid contents and stronger antioxidant activity, with an SC_50_ of 159 μg/mL for the DPPH assay. In contrast, FLE demonstrated more pronounced anti-inflammatory activity. In LPS-stimulated RAW 264.7 macrophages, FLE significantly reduced nitric oxide production and iNOS expression at both the protein and mRNA levels. FLE also reduced IL-6 secretion in a dose-dependent manner without affecting TNF-α, suggesting selective cytokine modulation. Both extracts exhibited low cytotoxicity (IC_20_ > 800 µg/mL in most cell types), non-hemolytic properties, and no mutagenic activity in the *Drosophila* wing spot assay. Compared to root and latex extracts, FLE ranked second in anti-inflammatory potency (FRE > FLE > FLA = FLtA). FLE, therefore represents a promising candidate, combining potent bioactivity with environmental responsibility and supporting the further development of *F. lindsayana* leaf-derived products for use in functional foods or botanical therapeutics.

## 1. Introduction

Inflammation is a crucial physiological mechanism serving as the first line of host defense against various harmful stimuli such as infections (viruses and bacteria), toxins, and physical injury. This complex immunovascular response preserves tissue homeostasis and integrity through a careful balance between protection and repair [1]. Macrophages play a pivotal role in regulating this process as they coordinate both the initiation and resolution of inflammation [2,3]. These innate immune cells regulate pro- and anti-inflammatory pathways, synthesize key mediators, and eliminate pathogens via phagocytosis. Major inflammatory mediators released by macrophages include cytokines such as tumor necrosis factor-alpha (TNF-α), interleukin-1β (IL-1β), and interleukin-6 (IL-6). These cytokines can activate enzymes such as inducible nitric oxide synthase (iNOS), which produces nitric oxide (NO), a signaling molecule involved in vasodilation, tissue damage, pain, and protein activation during inflammation [3,4]. Additionally, cytokines activate intracellular signaling pathways, including nuclear factor kappa B (NF-κB), mitogen-activated protein kinases (MAPKs), and phosphoinositide 3-kinase/protein kinase B (PI3K/AKT), which further modulate the inflammatory response [5,6,7].

While inflammation is essential for the body’s defense against tissue damage and infection, chronic or unresolved inflammation can lead to a range of diseases, including cardiovascular conditions, diabetes, cancer, and neurological disorders [7,8,9]. Chronic inflammatory disorders are a primary cause of mortality worldwide, responsible for approximately half of all deaths [9]. Epidemiological evidence links increased risks of cardiovascular disease and type 2 diabetes mellitus to both localized and systemic chronic inflammation, with the risk often correlating with the severity and duration of the inflammatory response [8]. Conventional treatment commonly employs nonsteroidal anti-inflammatory drugs (NSAIDs), which inhibit the cyclooxygenase (COX) enzyme to reduce prostaglandin synthesis and inflammation [10]. Nonetheless, NSAIDs are linked to significant adverse effects, particularly on the gastrointestinal and renal systems [10,11]. Even short-term NSAID use can increase the risk of gastrointestinal bleeding, myocardial infarction, and stroke. Furthermore, NSAID exposure is associated with acute kidney injury (AKI), tubulointerstitial nephritis (TIN), nephrotic syndrome, and progression to chronic kidney disease [10]. In adults aged over 65, NSAID use can more than quadruple the risk of AKI within 30 days, mostly due to inhibited prostaglandin synthesis essential for renal blood flow, particularly prostaglandin E_2_ (PGE2) and prostaglandin I2 (PGI2). In recent years, there has been a growing focus on utilizing natural products as alternative or adjunct therapy to prevent and manage inflammation-related diseases [12,13]. Plant-derived compounds such as curcumin, resveratrol, and capsaicin exhibit anti-inflammatory properties by modulating key signaling pathways, including COX, nitric oxide synthase (NOS), and NF-κB, as well as by reducing oxidative stress and suppressing pro-inflammatory cytokines.

The genus *Ficus*, one of the most taxonomically diverse and ecologically significant genera of flowering plants, includes more than 800 species primarily found in tropical and subtropical climates. Numerous *Ficus* species have been historically utilized in traditional medicine across Asia, Africa, and other regions for treating ailments such as inflammation, diabetes, and respiratory and gastrointestinal disorders [14,15,16]. For example, *Ficus benghalensis*, *Ficus religiosa*, and *Ficus elastica* have notable pharmacological effects, while *Ficus carica* is well recognized for its antibacterial, anticancer, and anti-inflammatory characteristics [17,18,19,20,21]. Phytochemical analyses indicate that *Ficus* species are rich in bioactive compounds, including phenolics, flavonoids, alkaloids, triterpenoids, and saponins, which are linked to antioxidant, antidiabetic, anticancer, hepatoprotective, and immunomodulatory effects [17,18]. Recently, interest has grown in lesser-known species such as *Ficus lindsayana* (formerly *Ficus dubia*), native to Southeast Asia, including Thailand, Malaysia, Indonesia, and Brunei. Extracts from its roots (FRE) and latex (FLtA) demonstrate strong antioxidant, anti-inflammatory, antiproliferative, and antidiabetic effects, largely due to high levels of phenolic and flavonoid components like caffeic acid and cyanidin [19,20,21]. FRE significantly suppresses lipopolysaccharide (LPS)-induced inflammation by downregulating iNOS, COX-2, IL-1β, IL-6, and TNF-α gene expression in macrophages [21]. Moreover, these extracts inhibit enzymes involved in diabetes, hypertension, and Alzheimer’s disease (AD) [22]. However, harvesting roots and latex for extracting these bioactive substances raises sustainability issues, as it can damage or destroy the plant and threaten biodiversity, thereby limiting their widespread use as a functional ingredient. In contrast, leaves provide a renewable and non-destructive source of pharmacologically active compounds, consistent with principles of sustainable natural resource management.

This study therefore aimed to comprehensively characterize the phytochemical metabolome, biological activities, including antioxidant and anti-inflammatory effects, and genotoxicity of *F. lindsayana* leaves (FL) in vivo. By comparing the bioactive profiles and functional properties of leaf extracts with those from previously studied root and latex [21], we provide critical evidence that leaves may serve as an equally potent yet more sustainable source for medicinal applications. Our findings provide practical recommendations for sustainable harvesting methods and guide the formulation of FL-derived products that maintain therapeutic efficacy while ensuring the conservation of plant populations.

## 2. Results

### 2.1. Extraction Yield, Total Phenolic Content, and Total Flavonoid Content of FLA and FLE

Extraction of *F. lindsayana* leaves (FL) using hot water and 80% ethanol produced dry extract yields of 32.9% (FLA) and 11.35% (FLE), respectively. The total phenolic content (TPC) was 141.65 ± 1.57 mg GAE/g for FLA and 38.52 ± 1.70 mg GAE/g for FLE (Table 1). The total flavonoid content (TFC) was also higher in FLA at 52.88 ± 1.08 mg CE/g, compared to 10.34 ± 1.38 mg CE/g in FLE (Table 1). These results demonstrate that hot water extraction is more effective than ethanolic extraction for isolating phenolic and flavonoid compounds from *F. lindsayana* leaves.

### 2.2. Comparative Metabolite Profiling of FLA and FLE

The Folin–Ciocalteu reagent used for TPC measurement in this study also reacts with non-phenolic reducing substances in plant extracts (e.g., ascorbic acid, certain amino acids, sugars, and organic acids). This reaction can significantly overestimate TPC. The Folin–Ciocalteu method estimated the total reducing capacity of the extracts, whereas HPLC-qTOF-MS profiling specifically maps phenolic composition. This specificity minimizes interferences and enables compound-level identification [23,24]. Comprehensive metabolomic profiling by HPLC-qTOF-MS (matching score > 95%) revealed that the FLA and FLE extracts possess distinct metabolite profiles, indicating significant differences in chemical diversity and abundance. FLA extract contained higher levels of polar, water-soluble metabolites, with chlorogenic acid (15.90%) as the most abundant phenolic acid. The dominant lipid-related compound was erucamide (28.51%), followed by 1-stearoyl-rac-glycerol (13.78%) and arachidonoylthiophosphorylcholine (7.07%) (Table 2). Notable nitrogenous metabolites included trigonelline (11.58%), adenine, and pantothenic acid (vitamin B5, 6.33%). Additional components included various amino acids (e.g., proline, isoleucine), nucleosides (adenosine), flavonoids (daidzein), and phospholipids.

The FLE extract exhibited greater chemical diversity, characterized by a pronounced presence of flavonoids and glycosides. These included rutin (0.92%), naringenin (0.74%), isorhamnetin 3-neohesperidoside, and kaempferol-3-O-rutinoside. High levels of chlorogenic acid were also observed, accounting for approximately 10.66%. The metabolite profile comprised a broad array of amino acids (proline, isoleucine, L-tryptophan), alkaloids (trigonelline 10.93%), nucleobases (adenine 5.71%), and vitamins (pantothenic acid, vitamins B2 and B6). Multiple lipid species were identified, with 1-palmitoyl-sn-glycero-3-phosphocholine (18.41%) and monolinolenin (3.74%) as the most abundant. Other prominent constituents included pomiferin, cinnamaldehyde, benzyldodecyldimethylammonium cation, and sterols (campesterol, friedelin). The fatty acid derivative 9-oxo-10E,12Z-octadecadienoic acid (14.87%), was detected exclusively in FLE (Table 3). Both extracts shared several major metabolites (e.g., chlorogenic acid, trigonelline, adenine, pantothenic acid). In contrast, FLA contained a higher proportion of hydrophilic compounds, notably erucamide and glycerolipids.

Compared to FLA, FLE exhibited greater chemical diversity, including a broader range of ethanol-soluble flavonoids, unique glycosides, fatty acids, and sterols. This diversity may contribute to the enhanced anti-inflammatory activity observed in bioassays. The identification of secondary metabolites unique to FLE, such as pomiferin and complex lipids, highlights the importance of solvent selection in isolating pathway-specific bioactive compounds. Overall, these results indicate that water and ethanol extracts of FL share several major metabolites but also possess distinct profiles, each potentially contributing unique pharmacological properties.

### 2.3. Phytochemical Composition of FLA and FLE Identified by LC-MS/MS and HPLC

The phytochemical profiles of FLA and FLE were analyzed using LC-MS/MS and HPLC techniques. LC-MS/MS identified chlorogenic acid, 3,4-dihydroxybenzoic acid, gallic acid, apigenin, rutin, and naringenin in FLE. In contrast, FLA contained a similar set of compounds, with caffeic acid detected instead of luteolin (Table 4). Chlorogenic acid was the most abundant compound in both extracts. These findings indicate that extraction solvent selection significantly affects the phenolic composition of FL leaf extracts. Hot water extraction (FLA) promoted the recovery of hydrophilic compounds, particularly caffeoylquinic acid derivatives and catechin, whereas ethanolic extraction (FLE) resulted in higher concentrations of luteolin and rutin. Both aqueous and ethanol extractions produced key bioactive compounds with known anti-inflammatory properties; however, each method selectively enriched specific phenolic constituents.

The HPLC chromatographic profiles of the two extracts differed, indicating compositional variation between the two extraction methods (Table 5). FLA contained higher concentrations of caffeoylquinic acid derivatives, with 3-caffeoylquinic acid (3-CQA), 4-caffeoylquinic acid (4-CQA), and 5-caffeoylquinic acid (5-CQA) measured at 7.32 ± 0.08, 9.92 ± 0.50, and 7.16 ± 0.20 mg/g extract, respectively. In FLE, these compounds were detected at lower concentrations: 3-CQA and 5-CQA at 3.37 ± 0.11 and 3.59 ± 0.02 mg/g extract, while 4-CQA was not detected. Catechin was also more abundant in FLA (6.00 ± 0.01 mg/g) compared to FLE (1.08 ± 0.02 mg/g).

### 2.4. Antioxidant Activities of FLA and FLE

**Figure 1:**
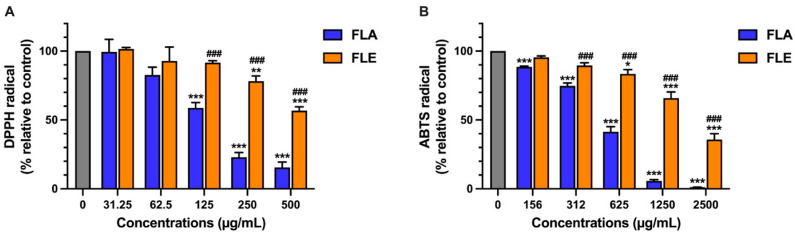
Antioxidant Activity of FLA and FLE by DPPH (**A**) and ABTS Assays (**B**). The data are indicated as mean ± SD of three independent experiments * *p* < 0.05, ** *p* < 0.01, and *** *p* < 0.001, against control. ### *p* < 0.001 compared to FLA at each concentration.

The antioxidant capacities of FL extracts were evaluated using DPPH and ABTS^•+^ radical scavenging assays. Both FLA and FLE exhibited dose-dependent radical scavenging activity, with FLA demonstrating significantly greater potency in both assays. The SC_50_ values for FLA were 159 µg/mL in the DPPH assay and 597 µg/mL in the ABTS assay (Figure 1A,B). In comparison, FLE showed lower activity, with an SC_50_ value exceeding 500 µg/mL for DPPH and 1989 µg/mL for ABTS^•+^.

The data indicate that the majority of antioxidant compounds in FL are more effectively extracted with hot water than ethanol, likely due to their hydrophilic nature. This finding is consistent with the previous measurement of higher TPC and TFC in FLA. The strong antioxidant activity of FLA support further investigation of its anti-inflammatory effects, its biological models of inflammation, specifically in LPS-stimulated RAW 264.7 macrophages.

### 2.5. Cytotoxicity Assessment of FLA and FLE

The cytotoxic effects of FLA and FLE were evaluated using a panel of mammalian cell lines: mouse mature 3T3-L1 adipocytes, preadipocytes (pre-3T3-L1), C2C12 myoblasts, RAW 264.7 macrophages, and human embryonic kidney (HEK 293) cells.

**Figure 2:**
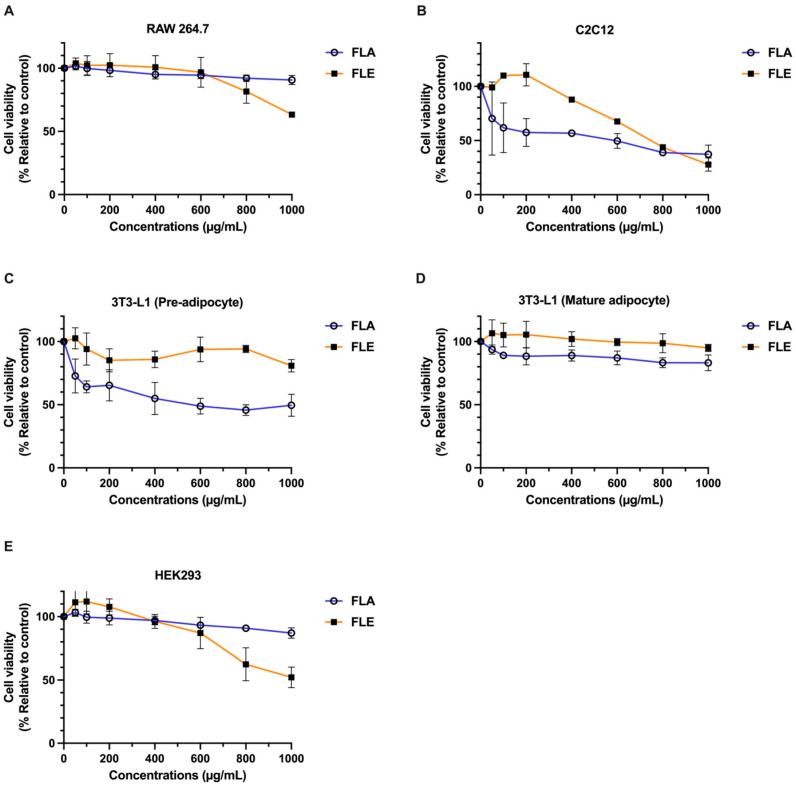
Cytotoxicity of FLA and FLE in Various Cells Determined by SRB Assay. RAW 264.7 (**A**), C2C12 (**B**), Premature 3T3-L1 (**C**), Mature 3T3-L1 (**D**), and HEK 293 cell (**E**). The data are indicated as mean ± SD of three independent experiments.

Both extracts exhibited low cytotoxicity in mature adipocytes, RAW 264.7 macrophages, and HEK 293 cells. In these models, IC_20_ values exceeded 800 µg/mL, and IC_50_ values were greater than 1000 µg/mL, indicating high cell viability (Figure 2). In contrast, more sensitive cell types exhibited distinct responses. FLE induced mild cytotoxicity in C2C12 myoblasts, with IC_20_ values between 400–600 µg/mL and IC_50_ values between 600–800 µg/mL. FLE remained non-toxic to pre-3T3-L1 adipocytes (IC_20_ > 1000 µg/mL). Conversely, FLA produced greater cytotoxic effects in both C2C12 myoblasts and pre-3T3-L1 adipocytes, with IC_20_ values below 50 µg/mL. However, IC_50_ values remained above 600 µg/mL in most cell types.

These results indicate that both FLA and FLE are safe for most cell types at concentrations below 800 µg/mL. FLA exhibited higher cytotoxicity in preadipocytes and myoblasts compared to FLE, suggesting that specific bioactive constituents in the aqueous extract may interact differently with distinct cell types. These results emphasize the importance of selecting a suitable cell model when evaluating botanical extracts for therapeutic or nutraceutical applications. The non-toxic concentrations of FLA and FLE extracts between 0–200 mg/mL were used in subsequent experiments.

### 2.6. Hemolytic Activity of FLA and FLE in Human Red Blood Cells (hRBCs)

The membrane-disruptive potential and biocompatibility of FL leaf extracts were assessed by a hemolysis assay using human red blood cells (hRBCs). Triton X-100 (1%) served as the positive control (representing 100% hemolysis), while untreated cells were used as the negative control. As shown in Table 6, both FLA and FLE exhibited minimal hemolytic activity at all tested concentrations (50–800 µg/mL). Hemolysis for both extracts remained below 2% at the highest concentration (800 µg/mL).

At 600 and 800 µg/mL, FLA resulted in 1.23% and 0.76% hemolysis, respectively, while FLE produced 0.32% and 0.10% hemolysis at the same concentrations. No detectable hemolysis (ND) was observed at 0 µg/mL. Based on established classification criteria, hemolysis of 0–10% is considered non-hemolytic, indicating that both extracts are hemocompatible at all tested doses. These results indicate that both FLA and FLE maintain red blood cell membrane integrity and may be suitable for oral or injectable formulations without inducing hemolysis-related cytotoxicity.

### 2.7. Mutagenicity Assessment of FLA and FLE in High Bioactivation Drosophila melanogaster

The potential mutagenic effects of FLA and FLE were evaluated using the in vivo somatic mutation and recombination test (SMART) in high bioactivation strains of *D. melanogaster*, which models the mammalian bioactivation system [25,26]. Mutant wing spot frequencies were measured at concentrations ranging from 62.5–5000 µg/mL. As summarized in Table 7, the negative control (DI) produced a background mutant frequency of 0.45 spots per individual, consisting solely of small single spots. No large or twin spots were observed in this group. The positive control (urethane, 20 mM) resulted in a total mutant spot frequency of 23.90 spots per individual, with significant increases in all spot categories. This outcome confirmed the assay’s sensitivity. For all tested concentrations of FLA, the total mutant spot frequency ranged from 0.38 to 0.75 spots per individual, predominantly as small single spots. Large single spots appeared sporadically at 250 and 5000 µg/mL, but no consistent pattern was observed. FLE treatments produced total mutant spot frequencies ranging from 0.28 to 0.53 spots per individual, with most groups lacking large or twin spots. Statistical diagnosis indicated that spot frequencies for both FLA and FLE at all concentrations were either equivalent to (−) or inconclusive (i) relative to the negative control. None of the treatments produced a mutagenic response comparable to the positive control.

The results indicate that neither FLA nor FLE caused a statistically significant increase in somatic mutant spots in high-bioactivation flies compared to the negative control, even at the highest tested concentration of 5000 µg/mL. Intermediate concentrations classified as “inconclusive” generally remained within the range of spontaneous background mutation. The pronounced mutagenicity observed with the positive control confirms the assay’s reliability. Therefore, FLA and FLE are considered non-mutagenic in this in vivo model, supporting their preliminary safety for further development as natural product ingredients.

### 2.8. Screening of Anti-Inflammatory Activity of FLA and FLE in LPS-Stimulated Macrophages

**Figure 3:**
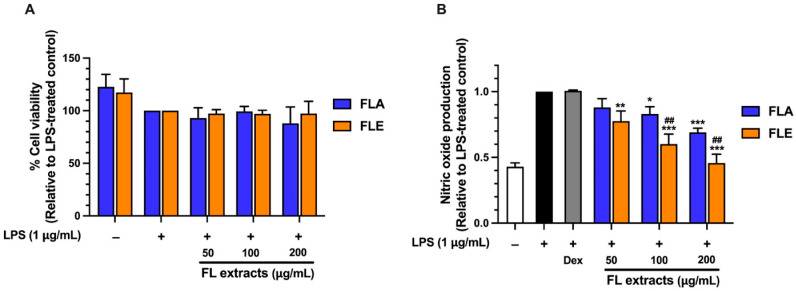
Effect of FLA and FLE Inhibits Nitric Oxide (NO) Production. Cell viability of co-treated LPS and the extracts (**A**). Nitric oxide production (**B**). The cells were treated with FLA and FLE for one hour, and then 1 μg/mL of LPS was added and further incubated for 24 h. After that, the treated cells were collected to determine cell viability by SRB assay, and the culture medium was subjected to determine NO production by Griess reagent. The data are indicated as mean ± SD of three independent experiments * *p* < 0.05, ** *p* < 0.01, and *** *p* < 0.001, against LPS-treated control (without the extracts). ## *p* < 0.01, compared to FLA at each concentration.

The anti-inflammatory potential of FL extracts was evaluated by measuring their ability to inhibit nitric oxide (NO) production in lipopolysaccharide (LPS)-stimulated RAW 264.7 macrophages. NO is a key pro-inflammatory mediator that plays a critical role in the inflammatory response and serves as a biomarker for the preliminary screening of anti-inflammatory compounds. FLA treatment resulted in significant, concentration-dependent suppression of NO production. At concentrations of 100 and 200 µg/mL, FLA reduced NO levels by 20% and 30%, respectively (Figure 3). In comparison, FLE exhibited a more potent inhibitory effect, reducing NO levels by 10%, 30%, and 50% at 50, 100, and 200 µg/mL, respectively (Figure 3). The greater dose-dependent inhibition observed with FLE suggests that this extract exhibits stronger anti-inflammatory activity in the LPS-stimulated macrophage model.

Although FLA contained significantly higher levels of TPC and TFC with greater antioxidant capacity, its anti-inflammatory activity was lower than that of FLE. This apparent discrepancy highlights the distinct mechanisms underlying antioxidant and anti-inflammatory effects. Phenolics and flavonoids contribute substantially to radical-scavenging activity (as shown in Figure 1A,B). In contrast, anti-inflammatory activity typically involves the regulation of cellular signaling pathways such as NF-κB and iNOS. These pathways may be more effectively modulated by specific ethanol-soluble compounds present in FLE, such as certain flavonoid aglycones or other less polar phytochemicals that are not abundantly extracted by hot water.

The stronger anti-inflammatory effect observed with FLE suggests that ethanol extraction yields bioactive constituents effective in suppressing pro-inflammatory responses. These findings emphasize the critical role of extraction methods in shaping bioactive compound profiles and demonstrate that TPC or TFC alone does not reliably predict anti-inflammatory efficacy.

### 2.9. Effect of FLA and FLE on iNOS and COX-2 Expression, TNF-α and IL-6 Secretion in LPS-Treated RAW 264.7 Cells

**Figure 4:**
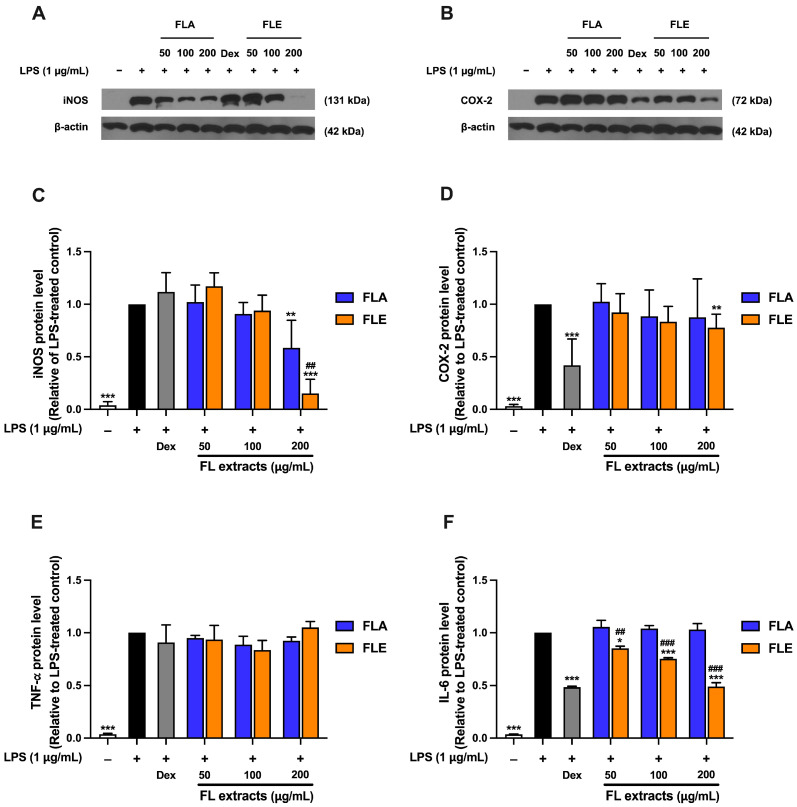
Effect of FLA and FLE on the Expression of Key Pro-Inflammatory Enzymes, iNOS and COX-2 (**A**–**D**), and the Key Pro-Inflammatory Cytokines, TNF-α and IL-6 (**E**,**F**). The protein expression of iNOS and COX-2 was determined using Western blotting. Band density and the protein level of iNOS (**A**,**C**). Band density and the protein level of COX-2 (**B**,**D**). Western blotting result of three independent experiments. Band density of the targeted protein normalized with β-actin levels. The pro-inflammatory cytokine secretion level was determined using ELISA. The protein secretion level of TNF-α (**E**) and IL-6 (**F**). RAW 264.7 macrophages were pre-treated with the extracts (0–200 µg/mL) for one hour, followed by 1 µg/mL LPS for 24 h. The treated cells were collected to determine protein expression. The supernatant was collected to examine the pro-inflammatory cytokine secretion level. Each value in (**C**–**F**) represents mean ± SD (*n* = 3) * *p* < 0.05, ** *p* < 0.01, and *** *p* < 0.001 vs. LPS-treated control. ## *p* < 0.01 and ### *p* < 0.001, compared to FLA at each indicated concentration.

To further elucidate the anti-inflammatory effects of FL extracts, the expression of two key pro-inflammatory proteins, inducible nitric oxide synthase (iNOS) and cyclooxygenase-2 (COX-2), was measured in LPS-stimulated RAW 264.7 macrophages. iNOS is responsible for NO production, and COX-2 catalyzes the synthesis of pro-inflammatory prostaglandins [27]. LPS stimulation significantly increased iNOS protein expression compared to the untreated (non-inflammatory) control group. Treatment with FLA or FLE at 200 µg/mL markedly reduced iNOS expression (Figure 4A,C), consistent with the observed decrease in NO production (Section 2.8). COX-2 expression was significantly increased following LPS stimulation (Figure 4B,D). FLE at 200 µg/mL produced a modest yet statistically significant suppression of COX-2 expression, whereas FLA did not affect COX-2 levels. These findings indicate that both extracts reduce NO production by downregulating the expression of iNOS protein, but only FLE partially modulates the COX-2 pathway, which synthesizes prostaglandin E_2_ (PGE_2_), a key mediator in inflammation and pain.

These findings demonstrate differences in the bioactive constituents of the two extracts. The suppression of both iNOS and COX-2 by FLE suggests greater effectiveness in modulating inflammatory signaling pathways compared to FLA. This observation supports the hypothesis that ethanol extracts contain unique or more potent anti-inflammatory compounds that are not efficiently extracted in water. To further investigate the impact on inflammatory response, the next subsequent experiment examined the effect of FLA and FLE on the secretion of pro-inflammatory cytokine in LPS-stimulated macrophages.

To further elucidate the anti-inflammatory mechanisms of FL extracts, the effects of FLA and FLE on the secretion of two key pro-inflammatory cytokines—tumor necrosis factor-alpha (TNF-α) and interleukin-6 (IL-6)—were assessed in LPS-stimulated RAW 264.7 macrophages. As shown in Figure 4E,F, LPS treatment significantly increased the secretion of both TNF-α and IL-6 compared to untreated control cells. Treatment with FLA did not significantly alter TNF-α or IL-6 levels. Similarly, FLE did not suppress TNF-α secretion at any of the tested concentrations (Figure 4E). In contrast, FLE treatment resulted in a dose-dependent inhibition of IL-6 secretion, with significant reductions of approximately 15%, 25%, and 50% at 50, 100, and 200 µg/mL, respectively (Figure 4F). These findings demonstrate that FLE and FLA exert anti-inflammatory effects through distinct molecular pathways. Both extracts reduce iNOS expression and inhibit nitric oxide production (Section 2.8). However, only FLE attenuates IL-6 secretion, which indicates pathway-selective regulation involving cytokine signaling. The absence of an effect on TNF-α suggests that FLE preferentially downregulates IL-6-associated transcriptional responses rather than broadly suppressing all inflammatory cytokines [28]. Taken together, these results indicate that the anti-inflammatory actions of FLA and FLE are mediated by distinct bioactive compounds that target specific components within the inflammatory response. This highlights the impact of the extraction solvent on the bioactivity profile.

**Figure 5:**
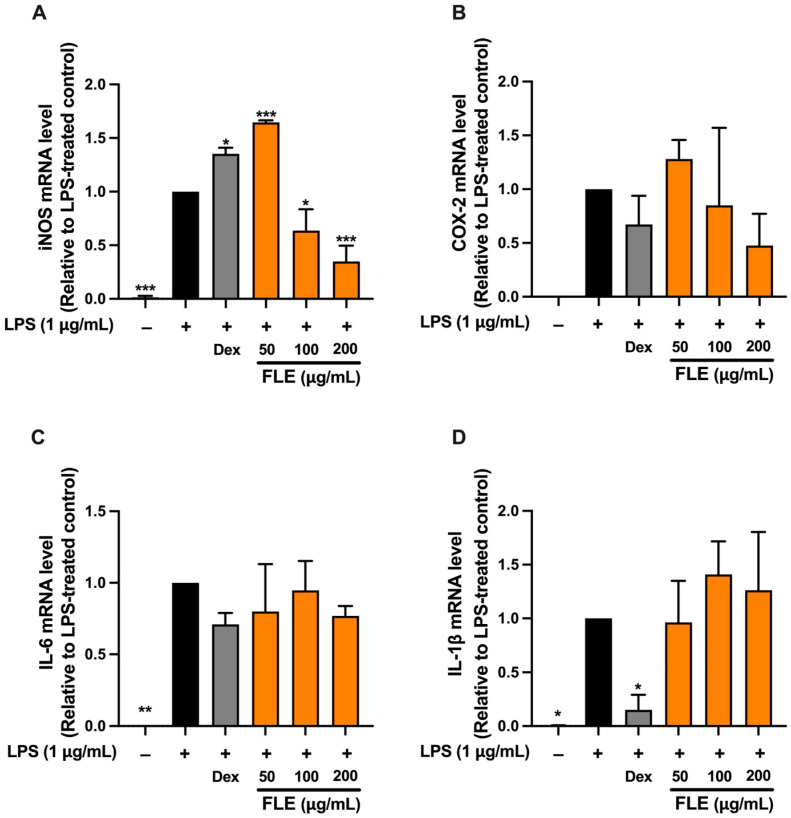
Effect of FLE on Key Pro-Inflammatory Enzyme mRNA Expression, iNOS (**A**) and COX–2 (**B**), and Pro-Inflammatory Cytokine mRNA Expression, IL–6 (**C**) and IL–1β (**D**). RAW 264.7 macrophages were pre-treated with FLE (0–200 µg/mL) for one hour, followed by 1 µg/mL LPS for 24 h. The treated cells were collected to determine mRNA expression of iNOS, COX–2, IL–6, and IL–1β by RT–qPCR, respectively. Each value represents mean ± SD (*n* = 3), * *p* < 0.05, ** *p* < 0.01 and *** *p* < 0.001 vs. LPS-treated group.

RT-qPCR analysis was subsequently performed only for FLE treatment, as FLE exhibited greater anti-inflammatory efficacy than FLA. The effects of FLE at concentrations of 50, 100, and 200 μg/mL on inflammatory gene expression were evaluated in LPS-induced RAW 264.7 macrophages. Dexamethasone (Dex), an anti-inflammatory agent, served as a positive control. LPS stimulation significantly increased mRNA expression of iNOS, COX-2, IL-6, and IL-1β compared to untreated controls (Figure 5). FLE at 100 and 200 μg/mL significantly reduced iNOS mRNA expression, with the greatest reduction at 200 μg/mL (*p* < 0.01), similar to the suppression observed with Dex (Figure 5). These data were consistent with the decrease in NO production and indicate that FLE exerts anti-inflammatory activity primarily by inhibition of the iNOS/NO pathway.

FLE administration did not result in a statistically significant reduction in COX-2 or IL-6 mRNA levels at any of the tested concentration. However, a non-significant downward trend was observed at 200 μg/mL for both targets. This observation aligns with Western blot analysis, which demonstrated that FLE at 200 μg/mL produced a slight but statistically significant reduction in COX-2 protein levels in LPS-stimulated macrophages. FLE treatment did not significantly alter IL-1β mRNA expression, whereas Dex significantly suppressed IL-1β mRNA compared to the LPS-only group (Figure 5). Despite the limited effect on IL-6 gene expression, FLE treatment significantly reduced IL-6 protein secretion into the culture medium.

Overall, the anti-inflammatory effect of FLE in LPS-stimulated RAW 264.7 macrophages is primarily mediated through the downregulation of iNOS expression and inhibition of nitric oxide production. The modest effects on COX-2 protein and the marked reduction in IL-6 secretion, despite the absence of significant mRNA suppression, suggest that FLE also influences post-transcriptional or post-translational regulatory mechanisms.

### 2.10. Network Pharmacology Analysis of Active Compounds Detected in FLA and FLE and Inflammation-Associated Genes

**Figure 6:**
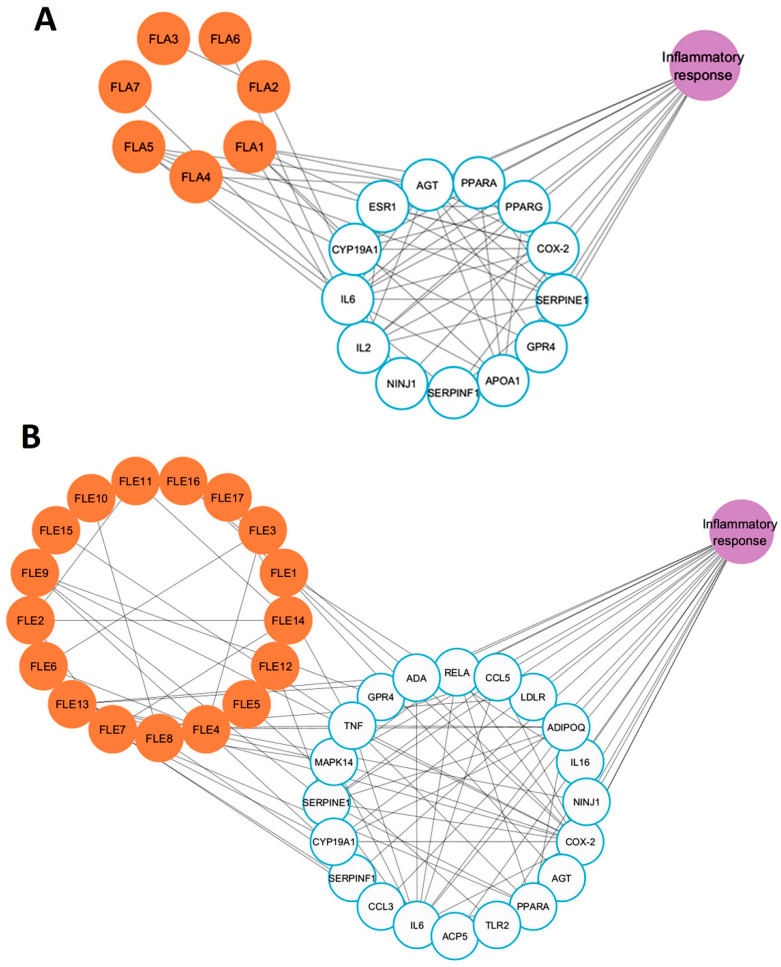
Network Pharmacology Analysis. The active compound of FLA and the inflammation-related gene network (**A**); the active compound of FLE and the inflammation-related gene network (**B**); Orange circles represent active compounds, and blue circles represent hub genes.

Since FLA exhibited weaker anti-inflammatory activity compared to FLE, a network pharmacology approach was used to elucidate the molecular basis of these findings. Metabolomic profiling, drug-likeness screening, target prediction, and network topology analysis were integrated to systematically compare the bioactive compound–target interactions of FLA and FLE with inflammation-associated genes. Due to the large number of compounds identified in the metabolomic analysis, compounds were initially screened for drug-likeness using Lipinski’s rule, a standard criterion in drug screening and design. Compounds meeting Lipinski’s criteria are generally associated with improved ADME (absorption, distribution, metabolism, and excretion) properties [29]. Based on these criteria, seven compounds from FLA and seventeen from FLE were selected for target gene analysis (Supplementary Tables S2 and S3). Comparison of FLA gene targets with inflammation-related genes from GeneCards and OMIM databases showed that seven FLA compounds (FLA1 to FLA7) interacted with thirteen inflammation-associated genes, including IL6, AGT, IL2, and NINJ1 (Figure 6A). In contrast, twenty inflammation-related genes, including ADA, LDLR, IL6, and CCL5, were found to interact with FLE compounds (FLE1 to FLE17) (Figure 6B).

The topological parameters of FLA1 to FLA7, and FLE1 to FLE17, specifically betweenness centrality and degree were analyzed. Betweenness centrality quantifies the frequency in which a node appears on the shortest paths between other nodes in the network, reflecting its role in connecting distinct network regions. A high betweenness centrality value indicates that the node behaves as a bridge or coordinator, facilitating efficient information exchange [30]. Degree refers to the number of direct connections a node makes to the rest. A high-degree node is often viewed as a hub due to their extensive connectivity [31].

Nodes exhibiting both high betweenness centrality and high degree are considered critical connectors and key participants within the network and are often prioritized for further study due to their potential biological significance. The betweenness centrality and degree values for key compounds in both FLA and FLE are presented in Table 8. The inflammation-related gene targets of FLA and FLE identified through the network are listed in Table 9.

The compound–target network of FLA was relatively sparse, which consisted of seven compounds and thirteen inflammation-related genes. The most highly connected compounds were 1-palmitoyl-sn-glycero-3-phosphocholine (betweenness = 0.0183; degree = 6) and daidzein (betweenness = 0.0074; degree = 6). Trigonelline, pantothenic acid, and 1-stearoyl-rac-glycerol had lower and minor betweenness centrality values (1–2), further showing small control in the network. In contrast, the FLE network presented a more complex structure with seventeen bioactive compounds associated with twenty inflammation-related genes. This configuration generated a denser web of interactions, suggesting a broader and more coordinated biological function. Within the FLE network, naringenin was identified as the most central node, which has the highest betweenness centrality (0.170) and degree (9), implying that naringenin is a pivotal hub and regulatory node. Chlorogenic acid also exhibited greater centrality in the FLE network compared to the FLA network. These findings indicate that FLE contains a diverse set of compounds capable of modulating key components of the inflammation signaling pathway.

The topology parameters derived from FLA target genes showed moderate connectivity. Key inflammation-related targets included SERPINE1, IL6, and AGT (degree = 11; betweenness = 0.05), as well as PPARG, PTGS2, and PPARA (degree = 9–11). Although these genes are known to participate in inflammatory pathways, their network centrality and regulatory influence were relatively modest. The NINJ1 gene exhibited low degree (2) and near-zero betweenness, indicating a marginal role within this network. In contrast, the FLE network displayed a larger and more cohesive inflammation-related target profile. PTGS2 (COX-2) showed the highest connectivity (betweenness = 0.0822; degree = 14), making it a central node in inflammatory signaling. Additional hubs included TNF (degree = 12) and IL6 (degree = 12), and MAPK14, RELA, and ADIPOQ (degree = 8–9). Further analyses identified several immune-regulating genes, including TNF, MAPK14, RELA, and TLR2, which were exclusively present in the FLE network. These genes function as upstream regulators of cytokine expression and immune cell activation, providing mechanistic insight into the potential of FLE as a broad-spectrum anti-inflammatory agent. In conclusion, the network pharmacology study demonstrates that FLE exhibits superior anti-inflammatory activity compared to FLA.

The network pharmacology analysis indicated that the ethanolic leaf extract (FLE) comprised a more extensive network of bioactive chemicals that target an expanded variety of inflammation-related genes. Significantly, key hub genes with elevated betweenness centrality and degree, including PTGS2 (COX-2), IL6, TNF, MAPK14, and RELA (NF-κB subunit), were recognized as central nodes within the FLE network. These genes are essential regulators of inflammatory signaling pathways, including the MAPK and NF-κB pathways. They participate in the regulation of pro-inflammatory enzymes iNOS and COX-2, as well as cytokines such as IL-6. The FLE network contains bioactive chemicals such as naringenin, which is recognized for its ability to influence these pathways. This supports the extract’s enhanced anti-inflammatory properties observed experimentally. This integrated analysis highlights the mechanistic basis by which FLE mediates regulation of inflammation through key pathway nodes.

### 2.11. A Comparative Study on the Anti-Inflammatory Activities of Aqueous (FLA), Ethanolic (FLE), Root (FRE), and Latex (FLtA) Extracts of F. lindsayana

**Figure 7:**
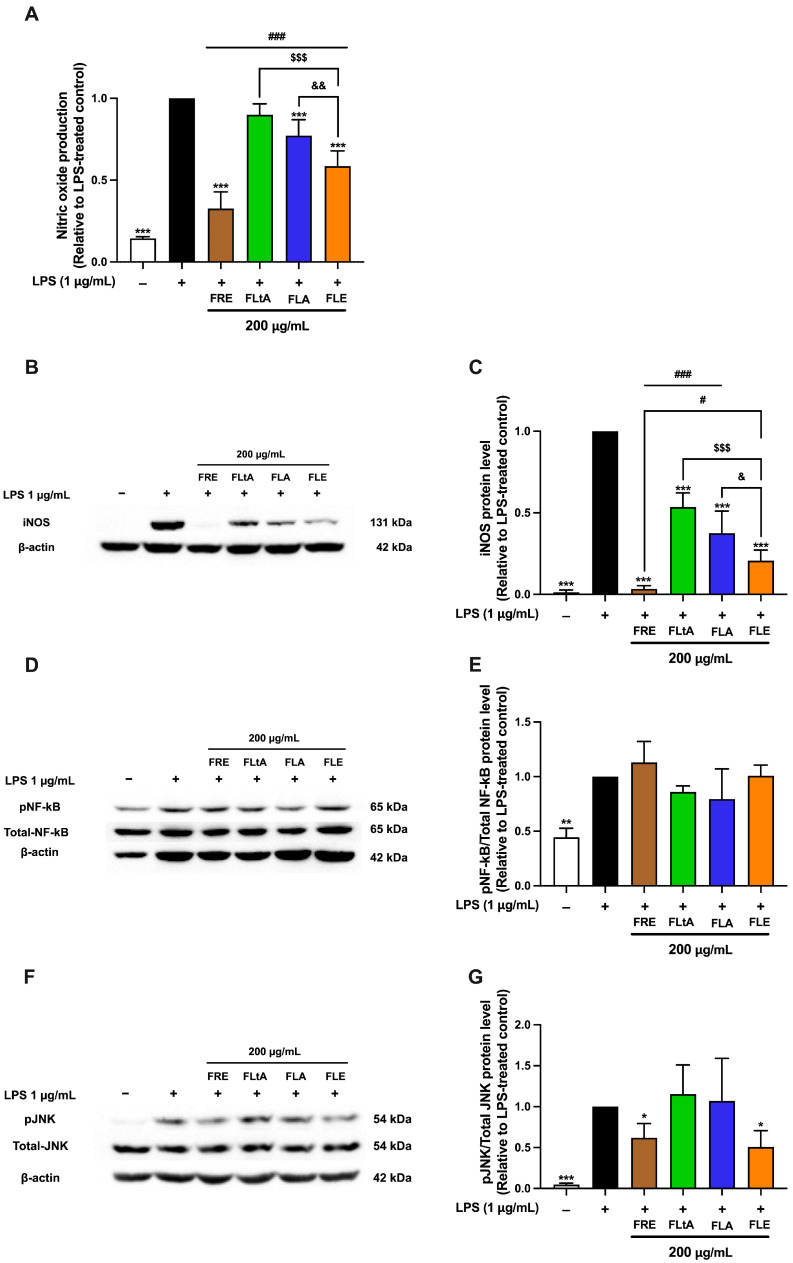
Anti-Inflammatory Activity of the Root, Latex, and Leaf Parts of *F. lindsayana* Extracts. The effect of FRE, FLtA, FLA, and FLE inhibits nitric oxide (NO) production (**A**). Each value represents mean ± SD (*n* = 3). The protein expression levels of iNOS, phosphorylated NF-κB (p-NF-κB), and phosphorylated JNK (p-JNK) were determined by Western blotting. Band densities corresponding to iNOS (**B**,**C**), p-NF-κB/total NF-κB (**D**,**E**), and p-JNK/total JNK (**F**,**G**) were quantified from three independent experiments. The band intensity of each target protein was normalized to β-actin or its respective total protein level. Each value represents mean ± SD (*n* = 3), * *p* < 0.05, ** *p* < 0.01, and *** *p* < 0.001 vs. LPS-treated group. # *p* < 0.05 and ### *p* < 0.001 vs. FRE. $$$ *p* < 0.001 FLtA vs. FLE. and & *p* < 0.05 and && *p* <0.01 FLA vs. FLE.

A comparative analysis of the anti-inflammatory activities of aqueous leaf (FLA), ethanolic leaf (FLE), root ethanolic (FRE), and latex aqueous (FLtA) extracts at 200 μg/mL revealed significant differences in efficacy across key inflammatory markers. Among all preparations, FRE exhibited the strongest inhibitory effects on nitric oxide production, iNOS protein expression, and phosphorylated c-Jun N-terminal kinase (phospho-JNK) levels, indicating superior anti-inflammatory potential (Figure 7). The phosphorylation of NF-κB was not significantly affected by any of the extracts. FLE consistently ranked second, with substantial inhibition of these mediators, although its effects were less pronounced than those of FRE. Both FLA and FLtA exhibited moderate inhibitory activity, with similar potency to each other but lower activity compared to FRE and FLE. The enhanced efficacy of FRE likely results from a higher concentration or greater diversity of anti-inflammatory constituents compared to both leaf- and latex-derived extracts. FLE represents a promising and more sustainable alternative to root-derived preparations, although it does not fully match the potency of FRE. These findings demonstrate the critical influence of extraction method and plant part selection in optimizing the therapeutic potential of *F. lindsayana*. Further investigation of FLE as an effective and ecologically responsible anti-inflammatory candidate is warranted.

## 3. Discussion

This study investigated the phytochemical diversity, bioactivity, and safety profiles of aqueous (FLA) and ethanolic (FLE) extracts of *F. lindsayana* leaf (FL). The integration of metabolomic, antioxidant, cytotoxicity, genotoxicity, network pharmacology, and mechanistic anti-inflammatory assays demonstrated that the ethanol extract of FL leaf represents a sustainable and promising alternative to conventional, ecologically harmful preparations of FL used in traditional medicine.

This study employed 80% ethanol and autoclave-assisted water extraction to target a broad spectrum of phytochemicals with minimal contamination and high efficiency. Hydroalcoholic extraction maximized the diversity and yield of metabolites, while autoclave-assisted water extraction facilitated the recovery of water-soluble and thermally stable compounds. This complementary strategy addressed the complex composition of plant matrices and met the requirements for both qualitative and quantitative phytochemical analyses [32,33]. Metabolomic and targeted HPLC and LC-MS/MS profiling revealed that solvent extraction has a significant impact on the chemical composition of FL extracts. The aqueous extract was enriched with hydrophilic constituents, such as chlorogenic acid, caffeoylquinic acid derivatives, catechin, pantothenic acid, and a range of amino acids. This finding is consistent with earlier reports on FL root and latex aqueous extracts, which also include chlorogenic acid, caffeic acid, and caffeoyl derivatives [18,19,20,21]. These metabolites are well-known effective antioxidants and contribute to several beneficial cellular functions [34,35,36]. In contrast, the ethanolic extract contains a broader range of phytochemicals. It includes glycosylated and aglycone flavonoids—notably rutin, naringenin, and luteolin—as well as specialized lipids, sterols, and intricate alkaloids. Our previous studies have shown that ethanolic extract of FL root (FRE) contains lower levels of phenolic acids, primarily chlorogenic acids (CGAs), but higher levels of flavonoids such as genistein, apigenin, naringenin, and luteolin [20].

Interestingly, rutin and naringenin identified in FLE exhibit well-documented activities against MAPK and NF-κB signaling in macrophages. Rutin has been reported to inhibit the release of NO and pro-inflammatory cytokines by suppressing the MAPK-JNK pathway, as well as other MAPKs and NF-κB signaling in activated macrophages. Naringenin, which is also found in FRE, attenuates both JNK and NF-κB phosphorylation and downstream inflammatory mediators [37,38,39,40,41,42]. Ethanol extraction increased the concentrations of rutin and naringenin in FLE, directly contributing to its ability to reduce JNK phosphorylation and downstream mediators such as iNOS and COX-2. The phytochemical composition provides a clear basis for the observed pathway specificity, supporting the mechanistic effects reported in this study. These observations support previous reports showing that extraction solvents influence plant-derived compounds’ yield, diversity, and bioactivity profiles [43,44,45,46], highlighting the need for careful solvent selection in natural product drug discovery.

FLA (141.65 ± 1.57 mg GAE/g extract and 52.88 ± 1.08 mg CE/g extract) demonstrated significantly higher TPC and TFC compared to FLE (38.52 ± 1.70 mg GAE/g extract and 10.34 ± 1.38 mg CE/g extract), leading to greater DPPH and ABTS radical scavenging activities. Notably, the TPC and TFC values of the leaf extracts (FLA and FLE) were lower than those previously reported for latex (248.53 ± 1.46 mg GAE/g extract and 55.78 ± 4.00 mg CE/g extract) and root extracts (208.31 ± 9.90 mg GAE/g extract and 157.33 ± 1.36 mg CE/g extract) from our group [20]. The antioxidant capacities were also consistent with this study, as the root extract exhibited higher DPPH and ABTS radical scavenging activities than the latex extracts [20]. This observation supports the role of phenolic acids and flavonoids as primary free radical neutralizers, as recognized in plant physiology and therapeutic applications. However, the relationship between antioxidant capacity and anti-inflammatory activity was not directly proportional. Similar discrepancies have been reported in studies of medicinal plants, where extracts with the highest antioxidant content do not always provide the most substantial reduction in inflammation [47]. These findings indicate that while antioxidant properties may protect against oxidative stress, effective inhibition of inflammatory signaling pathways requires targeted modulation of specific molecular mechanisms, such as iNOS/NO, COX-2, or cytokine activity.

Both FLA and FLE suppressed NO production and iNOS protein levels in LPS-stimulated RAW 264.7 macrophages, a standard method for evaluating anti-inflammatory efficacy. FLE had significantly stronger and dose-dependent effects, notably reducing IL-6 production and substantially decreasing COX-2 protein expression. Neither extract markedly influenced TNF-α or IL-1β, suggesting specificity in cytokine targeting and potential selectivity for the iNOS/IL-6 pathway. These findings agree with the activity of established anti-inflammatory phytochemicals and natural extracts, which often regulate iNOS/NO and select cytokines rather than universally inhibiting all inflammatory mediators [48,49,50]. Notably, FLE markedly reduced IL-6 protein secretion without a corresponding decrease in IL-6 mRNA, suggesting post-transcriptional regulation or modified cellular trafficking, as observed with other plant polyphenols (e.g., baicalin, glycyrrhizin) [51,52,53]. These findings highlight the importance of incorporating multi levels of analysis, including gene, protein, and functional markers, into screening techniques for the discovery of anti-inflammatory drugs.

A comparative analysis of different parts of *F. lindsayana* revealed that FRE most effectively suppressed key inflammatory mediators, including NO production, iNOS, and phosphorylated c-Jun N-terminal kinase (p-JNK). This finding aligns with previous studies in *Ficus* and other medicinal plant species, which indicates that roots typically contain a higher abundance or diversity of specialized metabolites responsible for anti-inflammatory activity [21,54]. Moreover, one of our previous studies reported that genistein, apigenin, chlorogenic acid, 3,4-dihydroxybenzoic acid, naringenin, luteolin, caffeic acid and gallic acid were detected in FRE in high amounts using LC-MS/MS analysis, whereas only chlorogenic acid and caffeic acid were detectable in FLtA—though at higher amounts than FRE [20].

Chlorogenic acid, 3,4-dihydroxybenzoic acid, and gallic acid were detected in both FLA and FLE, with lower amounts than in FLtA but higher than in FRE. Apigenin, rutin, luteolin, and naringenin were more abundant in FLE than in FLA, while FRE contained higher amounts of genistein, apigenin, luteolin and naringenin compared to both FLA and FLE. Notably, rutin was undetectable in FRE [20].

Moreover, the anti-inflammatory properties of these compounds, detected in all extracts, have been reported in LPS-induced RAW 264.7 cells through the inhibition of NO and pro-inflammatory cytokine production, and the NF-κB pathway [41,55,56,57,58]. However, systematically harvesting roots and latex is ecologically damaging and unsustainable [59,60]. In comparison, the ethanolic leaf extract (FLE), which ranked second in anti-inflammatory potency and diversity of specialized metabolites, also significantly inhibited these inflammatory markers. Furthermore, FLE offers advantages in sustainability, biodiversity preservation, and feasibility for large-scale production.

Mechanistically, the anti-inflammatory effects of FRE and FLE are primarily due to selective inhibition of the JNK signaling pathway. This action is not a result of direct modulation of the NF-κB pathway. FRE strongly inhibited p-JNK, NO, and iNOS expression. FLE had similar effects but were slightly less pronounced. Neither extract significantly affected NF-κB phosphorylation, indicating a mechanism that bypasses the canonical NF-κB inflammatory axis. In contrast, previous studies of aqueous latex extract demonstrated anti-inflammatory activity through NF-κB inhibition, particularly in cancer cell models [19]. The extract may exert specific inhibition of the JNK pathway in which a key MAPK regulates inflammatory gene expression via c-Jun phosphorylation and the AP-1 complex, leading to expression of genes such as iNOS and COX-2 [61,62]. Thus, selective targeting of JNK may enable effective control of inflammation and reduce immunosuppressive risks associated with NF-κB inhibition [63,64]. Still, future experiments should include AP-1 reporter assay, analysis of c-Jun nuclear translocation, and either pharmacologic or genetic JNK inhibition (e.g., with SP600125 or JNK siRNA) to demonstrate pathway dependence.

Several in vitro studies from RAW264.7 macrophage models support this mechanism, demonstrating that plant extracts inhibit JNK phosphorylation and downstream production of inflammatory mediators. For example, *Sambucus racemosa* subsp. *Pendula* leaf aqueous extract reduced NO, prostaglandin E2, iNOS, COX-2, and cytokines via toll-like receptor 4 (TLR4)-dependent JNK inhibition [65]. *Perilla frutescens* leaf extract suppressed LPS-induced phosphorylation of JNK and other MAPKs, lowering expression of iNOS, COX-2, cytokines, and nuclear translocation of NF-κB [66]. Methoxyflavonoids from *Kaempferia parviflora* also suppressed p-JNK and inflammatory markers in RAW264.7 cells [67].

A comprehensive safety evaluation demonstrated that both FLE and FLA are non-cytotoxic in standard mammalian cell lines, hemocompatible with less than 2% hemolysis at elevated dosages, and non-mutagenic in the *Drosophila* models that mimic mammalian bioactivation. These safety measurements contribute to the standardization of natural product development. However, the observed mild cytotoxicity of FLA in preadipocyte and myoblast cell lines indicates the need for further animal and clinical studies, especially for formulations intended for vulnerable populations such as children or the elderly.

Utilizing leaves, which are renewable and rapidly regenerating plant tissue, offer substantial ecological benefits. Sustainable leaf harvesting eliminates the need for destructive root or latex extraction, supporting the conservation of wild plant populations. This approach also complies with international standards for medicinal plant utilization established by the World Health Organization (WHO) and the International Union for Conservation of Nature (IUCN). Traditional Ficus-based remedies are associated with enhanced management of chronic inflammatory conditions, e.g., metabolic syndrome, diabetes, arthritis. Therefore, identifying a non-destructive, biochemically validated extract from Ficus leaves is crucial for advancing healthcare in both developed and developing countries.

Prior studies on Ficus latex and roots have documented their antioxidant, anti-tumor, anti-inflammation, and phytochemical profiles [18,19,20,21]. In comparison, the leaf extract demonstrates several distinct and advantageous attributes. First, it has a metabolite profile with considerable diversity, closely resembling the roots. This is particularly true for the range of specialized flavonoids and phenolic compounds associated with anti-inflammatory activity, though concentrations are generally lower. Second, the leaf extract exhibits selective anti-inflammatory effects, particularly through the inhibition of JNK. This contrasts with the broader cytotoxic or NF-κB suppression observed in some latex fractions. Finally, the leaf extract has a favorable safety and sustainability profile. It is non-cytotoxic at effective concentrations, and leaf harvesting is non-destructive and thus ecologically sustainable. These differences justify the focus on leaf extract as a sustainable and biochemically rich source for anti-inflammatory agents. They offer selective pathway modulation while enabling biodiversity preservation and scalable utilization.

Our study identified significant anti-inflammatory effects of FL extracts, specifically FLA and FLE in murine macrophage models. However, several limitations remain, including limited human relevance, uncharacterized active compounds, unknown pharmacokinetic profiles, and potential variability in extract composition. Future research should focus on isolating and characterizing bioactive constituents, elucidating mechanisms within inflammatory pathways, validating results in additional animal models, and conducting pharmacokinetic and safety assessments. Clinical trials are necessary to confirm the safety and efficacy of treatment in humans. Furthermore, implementing sustainable extraction methods and assessing the influence of harvest timing and geographic origin may improve extract consistency and quality. Comprehensive bioactivity screening and in vivo safety testing are essential to advance these extracts as standardized, effective, and safe anti-inflammatory agents.

## 4. Materials and Methods

### 4.1. Chemicals and Reagents

Calf serum (CS), Dulbecco’s Modified Eagle’s Medium (DMEM), penicillin/streptomycin, and trypsin-EDTA were purchased from Gibco (Grand Island, NY, USA). Fetal bovine serum (FBS) was supplied by Hyclone (Logan, UT, USA). Dexamethasone (Dex), 3-isobutyl-1-methylxanthine (IBMX), insulin, and lipopolysaccharide (LPS) were purchased from Sigma-Aldrich (St. Louis, MO, USA). Anti-COX-2, anti-JNK, and anti-phospho-JNK, anti-NF-κB p65, and anti-phospho-NF-κB p65 antibodies were obtained from Cell Signaling Technology (Danvers, MA, USA). Anti-iNOS and anti-β-actin antibodies were from Sigma-Aldrich (St. Louis, MO, USA). The Amersham™ Protran Premium 0.2 µm NC nitrocellulose blotting membrane was supplied by Cytiva (Cytiva, Dassel, Germany). Clarity Western ECL Substrate was purchased from Bio-Rad Laboratories (Hercules, CA, USA). HPLC standards for 3-caffeoylquinic acid, 4-caffeoylquinic acid, 5-caffeoylquinic acid, and catechin hydrate (all with HPLC purity > 98%) were acquired from Sigma-Aldrich (St. Louis, MO, USA). Standards for LC–ESI–MS/MS, including chlorogenic acid, 3,4-dihydroxybenzoic acid, caffeic acid, gallic acid, apigenin, rutin, naringenin, and luteolin (all with HPLC purity > 98%) were obtained from Tokyo Chemical Industry (Chuo-ku, Tokyo, Japan). TRIZol reagent was purchased from Invitrogen (Carlsbad, CA, USA). RevertAid RT Reverse Transcription Kit was obtained from Thermo Fisher Scientific (Waltham, MA, USA). SensiFAST™ SYBR Lo-ROX Kit was acquired from Bioline Ltd. (Little Clacton, Essex, UK). All primers for RT-qPCR were purchased from Bio Basic Inc. (Markham, ON, Canada).

### 4.2. Preparation and Extraction of F. lindsayana Leaf Samples

Fresh *F. lindsayana* leaves (FL) were collected and authenticated prior to processing. A voucher specimen (Chantarasuwan 040117-1) was deposited at the Thailand Natural History Museum (THNHM), Thailand Science Park, Pathum Thani, Thailand. The leaves were rinsed twice with distilled water, air-dried overnight, and then dried in a hot-air oven at 60 °C for 48 h. The dried material was ground into a fine powder.

To prepare the ethanolic leaf extract (FLE), powdered leaves were soaked in 80% ethanol at a 1:10 (*w*/*v*) ratio overnight with occasional stirring. This extraction process was repeated twice to maximize yield. The combined extracts were filtered, then concentrated under reduced pressure using a rotary evaporator, followed by freeze-drying with a lyophilizer.

To obtain the aqueous leaf extract (FLA), an autoclave-assisted extraction method was used. The powdered leaf sample was extracted with distilled water at a 1:10 (*w*/*v*) ratio in an autoclave [68]. The resulting extract was filtered and freeze-dried.

The yields of FLE and FLA were approximately 11.35% and 32.9% (*w*/*w*), respectively. All extracts were stored at –20 °C until further use.

The root ethanolic extract (FRE) and latex aqueous extract (FLtA) of FL were obtained from the remaining materials of a previously conducted study [21].

### 4.3. Determination of Total Phenolic Content (TPC) and Total Flavonoid Content (TFC)

TPC was measured using the Folin–Ciocalteu assay, following Budluang et al. (2017) [68]. Each extract was combined with Folin–Ciocalteu reagent and sodium carbonate, and the absorbance was recorded at 765 nm. Gallic acid and ferulic acid were used as reference standards. Results were expressed as milligrams of gallic acid equivalent (mg GAE/g extract) or ferulic acid equivalent (mg FAE/g extract) as calculated from the corresponding standard curves.

TFC was determined using the aluminum chloride (AlCl_3_) colorimetric method with minor modifications to the protocol described by Baba and Malik (2015) [69]. Briefly, each extract solution was mixed with AlCl_3_ and potassium acetate, then the absorbance was measured at 415 nm using a UV-Vis spectrophotometer. Catechin was used as the reference standard, and results were expressed as milligrams of catechin equivalents per gram of dry extract (mg CE/g extract), based on a standard calibration curve.

### 4.4. Non-Targeted Metabolomic Profiling by HPLC-qTOF-MS

Non-targeted metabolomic profiling of the aqueous (FLA) and ethanolic (FLE) extracts of FL was performed as described in the previous study [70], with slight modifications. Briefly, each sample was dissolved in 0.1% (*v*/*v*) dimethyl sulfoxide (DMSO) and filtered before injection.

Chromatographic separation was carried out using an Acclaim™ RSLC 120 C18 column (100 mm × 2.1 mm, 2.2 µm particle size, 120 Å pore size; Thermo Fisher Scientific, Waltham, MA, USA), coupled to a high-performance liquid chromatography–quadrupole time-of-flight mass spectrometry (HPLC-qTOF-MS) system (TripleTOF^®^ 6600; AB Sciex, Framingham, MA, USA). The mobile phase consisted of solvent A: 0.1% formic acid in water, and solvent B: 0.1% formic acid in acetonitrile. A linear gradient was applied over a 30 min run time at a flow rate of 0.4 mL/min. Mass spectrometric data were acquired in a positive ionization mode using full-scan acquisition across a mass range of 100–1000 *m*/*z*. Instrumental conditions were optimized for high-resolution data acquisition. The raw data were processed using SCIEX OS software, version 3.3.0 platform (AB Sciex). Metabolite identification was based on matching MS/MS spectra to entries in the High-Resolution MS/MS Spectral Library and the National Institute of Standards and Technology (NIST) database.

### 4.5. Targeted Phytochemical Analysis by LC-MS/MS

Liquid chromatography–tandem mass spectrometry (LC-MS/MS) was used to identify bioactive metabolites present in FLA and FLE. A high-resolution LC-MS/MS system with an electrospray ionization (ESI) source was operated in both positive and negative ion modes. Chromatographic separation utilized a C18 reversed-phase column under optimized gradient conditions with a binary solvent system: water containing 0.1% formic acid (Solvent A) and acetonitrile containing 0.1% formic acid (Solvent B). Flow rate, injection volume, and gradient program were optimized to maximize peak resolution and metabolite detection. Mass spectra were acquired in multiple reaction monitoring (MRM) and full-scan modes. Data analysis was performed using instrument-specific software. Metabolite identification was based on comparison of retention times and mass fragmentation patterns with authenticated reference standards and spectral databases.

### 4.6. Phytochemical Analysis by HPLC

The phytochemical profiles of the aqueous (FLA) and ethanolic (FLE) extracts of FL were analyzed using high-performance liquid chromatography (HPLC). The analysis was conducted on an Agilent Technologies HPLC system (Santa Clara, CA, USA) equipped with a C18 reversed-phase column (250 × 4.6 mm, 5 μm). The protocol for chlorogenic acid profile analysis was applied according to Craig AP et al. [71]. FLA and FLE were diluted in a mixing of 2.0% formic acid and 10% acetonitrile (ACN) to prepare a concentration of 20 mg/mL and then filtered through a 0.45 µM membrane filter. Trifluoroacetic acid (TFA) 0.3% in water and ACN were used as mobile phase A and B, respectively. The gradient program with a total run time of 40 min at a flow rate 1.0 mL/min. The gradient elution was as follows: 0–20 min, 95% to 80% A; 20–30 min, 80% to 5% A; 35–40 min, 95% to 5% A. The UV-Vis detector was set at 330 nm. In addition, to investigate catechin content in the extracts, the mobile phase consisted of solvent A (1% acetic acid in water) and solvent B (acetonitrile), applied in a gradient elution with a total run time of 50 min at a flow rate of 0.7 mL/min. The gradient program was as follows: 0–28 min, 90% to 60% A; 28–39 min, 60% to 40% A; 39–50 min, 40% to 10% A. Each extract was prepared at a concentration of 20 mg/mL in methanol, and 20 μL of the solution was injected into the column. Detection was performed at 280 nm using a UV-Vis detector. The chromatographic peaks were identified by comparing retention times with those of known reference standards, including 3-caffeoylquinic acid, 4-caffeoylquinic acid, and 5-caffeoylquinic acid for chlorogenic acid profile, and catechin hydrate, to determine the presence of specific phytochemical constituents.

### 4.7. DPPH and ABTS Assay

The antioxidant capacity of FL extracts was evaluated using DPPH (2,2-diphenyl-1-picrylhydrazyl) and ABTS [2,2′-azino-bis (3-ethylbenzothiazoline-6-sulfonic acid)] radical scavenging assays. The ABTS and DPPH assays were performed according to the method described by Budluang et al. (2017) and Karinchai et al. (2024) [21,68].

For both assays, the radical scavenging activity was calculated as the percentage of inhibition using the following equation:

(1) $$Radical\;scavenging\;activity\;(\%)=\frac{{({Abs}_{control}-Abs}_{extract})}{{Abs}_{control}}\times 100$$

where Abs_control_ is the absorbance of the control (without sample) and Abs_extract_ is the absorbance in the presence of the extract.

The results were expressed as SC_50_ values, indicating the concentration of extract (mg/mL) required to scavenge 50% of DPPH or ABTS radicals. SC_50_ values were determined from dose–response curves generated by plotting percent inhibition against sample concentration.

### 4.8. Cells and Cell Culture

Four normal cell lines were used in this study: C2C12 (mouse myoblasts, passages 5–10), HEK293 (human embryonic kidney cells, passage number ranged from 10 to 20), and 3T3-L1 (murine adipocytes, passages 5–10), RAW 264.7 (mouse macrophages, passages 30–40). These cell lines represent muscle, kidney, adipose tissue, and immune cells, respectively. All cell lines were obtained from the American Type Culture Collection (ATCC, Manassas, VA, USA). Cells were cultured in Dulbecco’s Modified Eagle Medium (DMEM) with L-glutamine supplement with 10% fetal bovine serum (FBS) (for C2C12, HEK293, 3T3-L1, and RAW 264.7) or 10% fetal calf serum (FCS) (for 3T3-L1) and 1% penicillin. Cultures were maintained at 37 °C in a humidified atmosphere with 5% CO_2_. Upon reaching 80% confluence, cells were harvested for subsequent experiments.

Human red blood cells (hRBCs) were isolated from blood samples of healthy individuals, as described by Karinchai et al. [21]. Samples were obtained from Maharaj Hospital, Chiang Mai, Thailand, anonymized by laboratory staff, and stored in heparinized tubes. The Research Ethics Committee of the Faculty of Medicine, Chiang Mai University (No. EXEMPTION 7561/2023), granted a certificate of exemption for this study. Consent to participate was not required due to the use of anonymous data. All procedures were carried out following relevant guidelines and regulations.

### 4.9. Cytotoxicity Testing

Cytotoxicity of FLA and FLE was examined in C2C12 (3000 cells/well), HEK293 (5000 cells/well), premature 3T3-L1 (3000 cells/well), and RAW 264.7 (25,000 cells/well). Cells were plated into a 96-well cell culture plate and incubated for 24 h. The cells were then treated with various concentrations of FLA or FLA (0–1000 µg/mL) for 48 h.

To assess effects in mature 3T3-L1 adipocytes, pre-3T3-L1 cells were seeded in 96-well plates (3000 cells/well) and incubated for two days. Differentiation was induced sequentially using induction media, differentiation media, and maturation media as previously described [21]. Upon achieving mature adipocyte status, cells were treated with various concentrations of FLA or FLE (0–1000 µg/mL) for 48 h.

At the indicated time, the cytotoxicity of FLA and FLE was determined by SRB assay as described previously [21]. The percent cell survival was calculated as follows:

(2) $$\%\;Cellular\;Viability=\frac{(Abs\;of\;treatment\;group)\times 100}{\left(Abs\;of\;control\;group\right)}$$

The inhibitory concentration of 20% (IC_20_) or a non-toxic concentration was used for further experiments.

### 4.10. Hemolysis Assay

A hemolysis assay was conducted to determine the hemolytic effect of FLA and FLE. In brief, human RBCs were collected by centrifuging whole blood samples. A 5% RBC suspension was prepared and combined with varying concentrations of the extracts, followed by incubation at 37 °C for three hours. Hemolysis was quantified according to a previously described method [21]. According to the guideline, less than 10% hemolysis is considered non-hemolytic while greater than 25% hemolysis is classified as highly hemolytic.

### 4.11. Genotoxicity Assessment of FLA and FLE in Drosophila

The mutagenicity of FLA and FLE was performed using the somatic mutation and recombination test (SMART) or wing spot test, following Inthachat et al. [25]. To assess the mutagenic potential of the extracts, 100 three-day-old larvae (third stage) were obtained and introduced to a *Drosophila* medium containing a non-toxic concentration of FLA or FLE. Urethane (20 mM) served as a mutagen control, whereas deionized water was utilized as a negative control. The larvae were fed on each medium until the pupation stage at 25 °C. Subsequently, the wings of the remaining flies had been taken for the analysis of mutant spots. Approximately 40 wings from each group were assessed for the presence of mutant spots, comprising small single spots, large single spots, and twin spots as described by Pitchakarn et al. [72]. The results were assessed by calculating estimates of spot frequencies and confidence limits for comparison with the control group, with statistical significance determined as previously stated by Frei and Würgler [73].

The *Drosophila* study was approved by the Institute of Nutrition-Mahidol University Institutional Animal Care and Use Committee (INMU-IACUC) (COA. No. INMU-IACUC, 2025/04).

### 4.12. Measurement of Nitric Oxide (NO)/Nitrite

The nitrite concentration in the cultured medium was measured using the Griess reaction [74,75]. RAW 264.7 macrophage cells were exposed to one-hour pretreatment with FLE and FLA at concentrations of 50, 100, 200 µg/mL. Following a 24 h incubation with LPS (1 µg/mL), the culture supernatant was collected for analysis using the Griess reagent assay [68]. NO production was calculated by comparison with the standard curve of sodium nitrite and analyzed in relation to cell survival rates.

### 4.13. Proinflammatory Cytokine Determination

After the treatment as described in Section 4.12, the level of TNF-α or IL-6 in the culture medium was quantified using a sandwich Enzyme-Linked Immuno-Sorbent Assay, carried out according to the manufacturer’s protocols (BioLegend’s ELISA MAXTM Deluxe Set, San Diego, CA, USA).

### 4.14. Immunoblotting

The treated cells were collected to investigate the expression of phospho-NF-kB p65 (Ser536), phospho-SAPK/JNK (Thr183/Tyr185), iNOS and COX-2. Protein samples were prepared, then subjected to 10% SDS-PAGE, and then the proteins were electrically transferred to a nitrocellulose membrane. The target proteins were probed with specific antibodies (anti-phospho-NF-kB p65 (Ser536) antibody at a dilution of 1:1000, anti-NF-kB p65 antibody at a dilution of 1:2000, anti-phospho-SAPK/JNK (Thr183/Tyr185) antibody at a dilution of 1:2000, anti-SAPK/JNK antibody at a dilution of 1:5000, anti-COX-2 antibody at a dilution of 1:2000, anti-iNOS antibody at a dilution of 1:2000, anti-β-actin antibody at a dilution of 1:10,000). Protein detection was carried out using a chemiluminescent reagent. The visible bands of phospho-NF-kB p65 (Ser536), NF-kB p65, phospho-SAPK/JNK, SAPK/JNK, iNOS, COX-2, and β-actin were shown at 65, 65, 54, 54, 131, 74, and 42 kDa, respectively.

### 4.15. mRNA Levels of Inflammatory Mediator Determined by Reverse Transcription-Quantitative Polymerase Chain Reaction (RT-qPCR)

Following the treatment, total RNA was extracted from the cells and measured using a nanodrop spectrophotometer. Subsequently, total RNA was subject to reverse transcription in order to generate cDNA. The mRNA level was quantified using quantitative reverse transcription polymerase chain reaction (RT-qPCR) using the specific primers listed in Table S1. The target mRNA amounts were normalized to the housekeeping gene mRNA levels, and the values obtained with or without the extracts were expressed in relation to the values derived from the control group.

### 4.16. Network Pharmacology

#### 4.16.1. Screening of Active Components and Prediction of Targets of FLA and FLE

The metabolomic data presented in Table 2 and Table 3 were used in this section. Chemical structure and SMILES formulas for compounds, excluding amino acids, were retrieved from the PubChem database. Before evaluating Lipinski’s rule, these structures were submitted to the Swiss ADME database. Compounds that met Lipinski’s criteria were selected and further screened for potential targets using the Swiss Target Prediction database and PharmMapper databases. After removing duplicate targets, the corresponding genes were identified via the UniProt database.

#### 4.16.2. Construction of Active Component-Target Network

The active compounds of FLA and FLE and their potential gene targets were analyzed in Cytoscape (version 3.10.3) to generate the active component-target network. The Network Analyzer tool was used to calculate topological parameters, and the component–target network was subsequently analyzed based on degree values. Subsequently, inflammation-related target genes were identified using the Human Genome Annotation (GeneCards) and OMIM databases with “inflammatory response” as the keywords. After removing duplicate entries, the corresponding genes were verified through the UniProt database.

#### 4.16.3. Generation of Protein–Protein Interaction (PPI) Network

The PPI network was constructed by identifying the overlapping targets between the active compounds and inflammation-related genes using the STRING database, with the interaction confidence score set to the highest level (0.9). The resulting PPI data were imported into Cytoscape for network analysis. Targets with degree values exceeding the median were selected for further visualization and construction of the PPI network.

### 4.17. Statistical Analysis

All values are shown as mean ± standard deviation (X ± SD) derived from triplicate samples across three different tests. The disparities between the two groups were examined by *t*-test analysis utilizing GraphPad Prism version 10.4.2 (GraphPad Software, Boston, MA, USA). Overall differences among the treatment groups were assessed utilizing a one-way analysis of variance (ANOVA), followed by Tukey’s multiple comparison test, employing GraphPad Prism version 10.4.2 (GraphPad Software, Boston, MA, USA). P-values less than 0.05 were considered indicative of statistical significance. The statistical analysis of the wing spot test was performed by Frei and Würgler (1988), utilizing significance thresholds of α = β = 0.05 [73].

## 5. Conclusions

This study investigates the chemical composition and biological activity of *F. lindsayana* leaf extracts obtained by autoclave-assisted water extraction (FLA) and ethanolic extraction (FLE). The FLA extract contains higher concentrations of phenolic and flavonoid compounds, exhibiting improved antioxidant ability. In contrast, the FLE extract showed significant anti-inflammatory effects by inhibiting nitric oxide production and regulating cytokine expression in macrophages. Both extracts demonstrated favorable safety profiles, characterized by a lack of cytotoxicity, hemolytic activity, and genotoxicity, which supports their potential for human use. The ethanolic leaf extract offers a sustainable alternative to root and latex derivatives, thereby reducing environmental impact while maintaining therapeutic efficacy. Further research is needed to identify the active constituents, confirm their mechanisms of action in vivo, and assess their clinical potential for incorporation into nutraceutical or herbal medicinal products.

## Figures and Tables

**Table 1:** Yield (%), Total Phenolic Content (TPC), and Total Flavonoid Content (TFC) of FLA and FLE.

	% Yield	TPC (mg GAE/g Extract)	TFC (mg CE/g Extract)
**FLA**	32.90	141.65 ± 1.57 ****	52.88 ± 1.08 ****
**FLE**	11.35	38.52 ± 1.70	10.34 ± 1.38

Results are shown as mean ± SD of triplicate experiments (*n* = 3). Statistical analysis was performed using an independent *t*-test to compare FLA with FLE. **** *p* < 0.0001 indicates statistically significant differences. GAE = gallic acid equivalent; CE = catechin equivalents.

**Table 2:** Metabolite Profiling of FLA as Determined by HPLC-qTOF-MS (Positive Mode-Cut Off Library Score 95).

NO.	RT (min)	Tentative Compounds	Adduct	MS/MS	Molecular Mass	Library Score	Relative Area (%)
1	25.59	Erucamide	M+	69.0703, 95.0857, 97.1014, 135.1171, 338.3476	337.34643	95.6	28.51
2	4.84	Chlorogenic acid	M+	163.0478, 355.1141, 356.1114	354.1068	99.5	15.90
3	23.37	1-Stearoyl-rac-glycerol	M+	57.0704, 71.0861, 95.0859, 359.3210	358.31417	95.9	13.78
4	1.00	Trigonelline	M+	136.0665, 138.0597, 139.0440	137.05296	99.3	11.58
5	26.66	Arachidonoylthiophosphorylcholine	M+	184.0756, 784.5909	783.58181	99.6	7.07
6	2.54	Pantothenic acid	M + H+	70.0295, 90.0559, 124.0766, 220.1271	219.12	95.6	6.33
7	1.31	Adenine	M+	65.0139, 92.0247, 92.0400, 109.0506, 119.0359, 136.0619	135.06088	97.7	5.40
8	8.61	Daidzein	M+	65.0386, 91.0540, 137.0233, 181.0646, 199.0756, 255.0655	254.0702	97.3	3.14
9	1.53	Isoleucine	M+	58.0501, 69.0705, 86.0971, 132.1080	131.10318	96.7	1.81
10	6.57	2-Linoleoylglycerol	M + Na+	71.0506, 193.0859, 377.2157	354.23067	100	1.65
11	0.98	Proline	M+	70.0655, 114.0955, 116.0749	115.06794	98.5	0.98
12	1.40	Adenosine	M+	136.0623, 209.8730	267.1087	100	0.94
13	23.19	Stearamide	M+	57.0702, 88.0760, 102.0915, 284.2963	283.29436	97.8	0.81
14	18.22	1-Palmitoyl-sn-glycero-3-phosphocholine	M+	86.0970, 104.1080, 184.0760, 496.3516	495.34665	99.6	0.79
15	6.32	Syringaldehyde	M+	65.0388, 77.0388, 95.0493, 140.0478	182.06424	97.4	0.61
16	7.45	1H-Indole-4-carboxaldehyde	M+	91.0563, 118.0662, 146.0659	145.05909	97.7	0.35
17	1.32	Nicotinic acid	M+	53.0390, 78.0344, 78.9855, 124.0396	123.04432	98.2	0.35

**Table 3:** Metabolite Profiling of FLE as Determined by HPLC-qTOF-MS (Positive Mode-Cut Off Library Score 95).

NO.	RT (min)	Tentative Compounds	Adduct	MS/MS	Molecular Mass	Library Score	Relative Area (%)
1	18.23	1-Palmitoyl-sn-glycero-3-phosphocholine	M+	86.0970, 104.1094, 184.0808, 496.3431	495.34665	99.6	18.41
2	17.22	9-Oxo-10E,12Z-octadecadienoic acid	M + H+	55.0544, 95.0856, 109.1011, 263.2377	294.22764	96	14.87
3	1.00	Trigonelline	M+	65.0386, 78.0341, 92.0496, 93.0560, 138.0552	137.05296	99.7	10.93
4	4.83	Chlorogenic acid	M+	117.0339, 145.0291, 163.0404	354.1068	99.5	10.66
5	2.52	Pantothenic acid	M + H+	60.0447, 72.0445, 98.0240, 124.0761, 202.1046	219.12	97.2	6.73
6	1.25	Adenine	M+	65.0138, 92.0248, 119.0359, 136.0623	135.06088	99.8	5.71
7	1.52	Isoleucine	M+	57.0581, 69.0710, 86.0973	131.10318	98.9	5.65
8	18.75	Monolinolenin	M+	81.0705, 107.0860, 121.1017, 149.1331, 173.1332, 261.2228	352.27471	98.3	3.74
9	7.45	1H-Indole-4-carboxaldehyde	M+	65.0389, 91.0547, 116.0498, 146.0863	145.05909	97.6	3.32
10	22.91	Friedelin	M+	21.0700, 109.1015, 137.1330, 427.3938	426.39071	97.1	2.49
11	24.95	Campesterol	M + H+	81.0703, 147.1172, 383.3681	382.36593	96.7	2.12
12	7.45	Indole	M+	65.0390, 91.0544, 118.9068	117.0635	96.4	1.74
13	4.06	L-Tryptophan	M + H+	91.0556, 115.0563, 118.0716, 146.0636	204.09646	95	1.46
14	10.64	9,12-Octadecadiynoic acid	M+	55.0544, 93.0699, 95.0856, 121.1018, 277.2134	276.21558	95.6	1.33
15	20.08	1-Monolinoleoyl-rac-glycerol	M + H+	81.0706, 95.0860, 109.1017, 263.2389, 337.2754	354.28415	95.5	1.33
16	1.00	Proline	M+	70.0662, 116.0724	115.06794	98.5	1.32
17	23.19	Stearamide	M+	57.0703, 88.0762, 116.1075, 284.2974	283.29436	96.9	1.04
18	16.75	Benzyldodecyldimethylammonium cation	M+	91.0542, 212.2374, 304.2999	303.29897	95.1	0.97
19	23.36	1-Stearoyl-rac-glycerol	M+	57.0704, 71.0859, 95.0857, 109.1014, 123.1171	358.31417	95.7	0.95
20	1.53	Vitamin B6	M+	77.0391, 134.0610, 152.0722	169.07994	98.7	0.94
21	6.56	Rutin	M+	85.0287, 303.0523	610.1687	97.2	0.92
22	9.92	Naringenin	M+	91.0542, 119.0492, 147.0445, 153.0194	272.07329	97.8	0.74
23	7.05	Aempferol-3-O-rutinoside	M+	85.0284, 287.0568	594.16768	97.6	0.52
24	5.55	Vitamin B2	M+	172.0876, 198.0657, 243.0874, 377.1474	376.14502	95.3	0.39
25	1.52	1,5-Diazabicyclo4.3.0-non-5-ene	M+	68.0499, 97.0765, 125.1082	124.02136	98.6	0.34
26	7.30	1H-Indole-3-carboxylic acid	M+	89.0389, 91.0545, 116.0498, 144.0447	144.02674	98.9	0.30
27	6.89	Cinnamaldehyde	M+	77.0387, 91.0543, 103.0536, 115.0541, 132.8992	132.09821	95.3	0.27
28	18.45	Pomiferin	M+	213.0559, 347.0935, 365.1039, 421.2428	420.16253	96.6	0.26
29	11.19	7.alpha.,24(S)-Dihydroxycholesterol	M+	365.2678, 383.2788	382.27446	100	0.21
30	4.97	2-Phenylbutyric acid	M + H+	65.0387, 91.0549, 119.0492, 164.0635	164.0528	96.4	0.14
31	7.16	Isorhamnetin 3-neohesperidoside	M+	85.0276, 317.0654, 625.2376	624.17356	95.2	0.10
32	6.57	Butaprost	M + Na+	111.0441, 139.0384, 377.2140	354.23067	96.2	0.09

**Table 4:** The quantitative phytochemical compounds in FLA and FLE, as determined by LC-MS/MS.

Compounds	(mg/g Extract)	
	FLA	FLE
Chlorogenic acid	1.07 ± 0.011	1.31 ± 0.043 *
3,4-Dihydroxybenzoic acid	0.40 ± 0.027	0.57 ± 0.016 *
Caffeic acid	0.23 ± 0.014	ND
Gallic acid	0.09 ± 0.002	0.24 ± 0.007 **
Apigenin	0.026 ± 0.001	0.077 ± 0.001 ***
Rutin	0.018 ± 0.003	0.068 ± 0.004 **
Naringenin	0.003 ± 0.003	0.055 ± 0.001 **
Luteolin	ND	0.065 ± 0.003

Results are shown as mean ± SD of triplicate experiments (*n* = 3). Statistical analysis was performed using an independent *t*-test to compare FLA with FLE. * *p* < 0.05, ** *p* < 0.01, and *** *p* < 0.001 indicates statistically significant differences. ND = Not detectable.

**Table 5:** Quantitative Phytochemical Compounds in FLA and FLE, as Determined by HPLC.

	Phenolic Acids (mg/g Extract)			Flavonoids (mg/g Extract)
	Chlorogenic Acids (CGAs)			Catechin
	3-CQA	4-CQA	5-CQA	
FLA	7.32 ± 0.08 ***	9.92 ± 0.50 **	7.16 ± 0.20 **	6.00 ± 0.01 ****
FLE	3.37 ± 0.11	ND	3.59 ± 0.02	1.08 ± 0.02

The result was expressed as mean ± SD, *n* = 3. Statistical analysis was performed using an independent *t*-test to compare FLA with FLE. ** *p* < 0.01, *** *p* < 0.001, and **** *p* < 0.0001 indicates statistically significant differences. ND = not detectable; 3-CQA, 3-caffeoylquinic acid; 4-CQA, 4-caffeoylquinic acid; 5-CQA, 5-caffeoylquinic acid.

**Table 6:** Hemolysis of FLA and FLE Extracts in hRBCs.

Concentration (μg/mL)	% Hemolysis	
	FLA	FLE
Triton X-100 (positive control)	100 ± 1.90	100 ± 1.89
0	ND	ND
50	0.56 ± 0.11	0.52 ± 0.21
100	0.56 ± 0.08	0.51 ± 0.29
200	0.35 ± 0.05	0.28 ± 0.06
400	0.54 ± 0.24	0.35 ± 0.15
600	1.23 ± 0.06	0.32 ± 0.09
800	0.76 ± 0.19	0.10 ± 0.00

The results are expressed as mean ± SD, *n* = 5. 0–10% hemolysis is classed as non-hemolysis, 10–25% hemolysis is slight hemolysis induction, and >25% hemolysis is high hemolysis induction. ND = not detectable.

**Table 7:** Mutagenicity of FLA and FLE extracts reported as wing spot induction in high bioactivation *Drosophila melanogaster*.

Samples		Frequency of Mutant Spots per Individual (Number of Spots) ^#^			
		Small Single (1–2 Cells)	Large Single (>2 Cells)	Twin	Total Spots
DI (negative control)		0.45 (18)	0.00 (0)	0.00 (0)	0.45 (18)
Urethane (Positive control)	20 mM	19.18 (767) +	3.28 (131) +	1.45 (58) +	23.90 (956) +
FLA	62.5 µg/mL	0.40 (16) −	0.00 (0) −	0.00 (0) −	0.40 (16) −
	125 µg/mL	0.55 (22) i	0.00 (0) −	0.00 (0) −	0.55 (22) i
	250 µg/mL	0.53 (21) i	0.03 (1) i	0.00 (0) −	0.55 (22) i
	500 µg/mL	0.53 (21) i	0.00 (0) −	0.00 (0) −	0.53 (21) i
	1000 µg/mL	0.50 (20) i	0.00 (0) −	0.00 (0) −	0.50 (20) i
	2000 µg/mL	0.38 (15) −	0.00 (0) −	0.00 (0) −	0.38 (15) −
	5000 µg/mL	0.70 (28) i	0.03 (1) i	0.00 (0) −	0.75 (29) i
FLE	62.5 µg/mL	0.45 (18) −	0.00 (0) −	0.00 (0) −	0.45 (18) −
	125 µg/mL	0.40 (16) −	0.00 (0) −	0.00 (0) −	0.40 (16) −
	250 µg/mL	0.48 (19) −	0.00 (0) −	0.00 (0) −	0.48 (19) −
	500 µg/mL	0.28 (11) −	0.00 (0) −	0.00 (0) −	0.28 (11) −
	1000 µg/mL	0.5 (21) i	0.00 (0) −	0.00 (0) −	0.53 (21) i
	2000 µg/mL	0.48 (19) −	0.00 (0) −	0.00 (0) −	0.48 (19) −
	5000 µg/mL	0.38 (15) −	0.00 (0) −	0.00 (0) −	0.38 (15) −

^#^ Statistical diagnoses using estimation of spot frequencies and confidence limits according to for comparison with DI (negative control); + = positive; − = negative; i = inconclusive. Probability levels: α = β = 0.05. One-sided statistical test “m” is an increased mutation frequency compared with the spontaneous frequency (m times).

**Table 8:** Topology Parameters of Important Compounds in FLA and FLE in the Network.

Display Name	Full Term	Betweenness Centrality	Degree
**FLA**			
FLA6	1-Palmitoyl-sn-glycero-3-phosphocholine	0.0183	6
FLA1	Daidzein	0.0074	6
FLA2	Chlorogenic acid	0.0076	2
FLA7	Erucamide	0.0023	1
FLA3	Trigonelline	0.0023	1
FLA5	1-Stearoyl-rac-glycerol	0.0026	1
FLA4	Pantothenic acid	0.0016	1
**FLE**			
FLE7	Naringenin	0.1700	9
FLE13	Cinnamaldehyde	0.0080	5
FLE14	Chlorogenic acid	0.0500	4
FLE9	1-Palmitoyl-sn-glycero-3-phosphocholine	0.0040	4
FLE1	Trigonelline	0.0230	3
FLE2	Rutin	0.0280	3
FLE11	Kaempferol-3-*O*-rutinoside	0.0020	2
FLE16	Adenine	0.0020	2
FLE3	Vitamin B2	0.0020	2
FLE4	Vitamin B6	0.0020	2
FLE6	Pantothenic acid	0.0020	2
FLE8	1-Monolinoleoyl-rac-glycerol	0.0620	2
FLE10	Monolinolenin	0.0020	1
FLE12	1-Stearoyl-rac-glycerol	0.0020	1
FLE17	9-Oxo-10E,12Z-octadecadienoic acid	0.0020	1
FLE5	Pomiferin	0.0020	1
FLE15	Campesterol	0.0020	0

**Table 9:** Topology Parameters of the Inflammation-Related Genes Targets of FLA and FLE in the Network.

Display Name	Full Term	Betweenness Centrality	Degree
**FLA**			
SERPINE1	Serpin Family E Member 1	0.0499	11
IL6	Interleukin 6	0.0321	11
AGT	Angiotensinogen	0.0499	11
PPARG	Peroxisome Proliferator-Activated Receptor Gamma	0.0262	11
ESR1	Estrogen Receptor 1	0.0209	10
PTGS2	Prostaglandin-Endoperoxide Synthase 2 (also known as COX-2)	0.0239	10
PPARA	Peroxisome Proliferator-Activated Receptor Alpha	0.0168	9
IL2	Interleukin 2	0.0146	8
APOA1	Apolipoprotein A1	0.0127	7
CYP19A1	Cytochrome P450 Family 19 Subfamily A Member 1 (Aromatase)	0.0096	6
GPR4	G Protein-Coupled Receptor 4	0.0049	3
SERPINF1	Serpin Family F Member 1	0.0049	3
NINJ1	Ninjurin 1	0.0015	2
**FLE**			
PTGS2	Prostaglandin-Endoperoxide Synthase 2 (COX-2)	0.0822	14
TNF	Tumor Necrosis Factor	0.0427	12
IL6	Interleukin 6	0.0236	12
SERPINE1	Serpin Family E Member 1	0.0138	9
ADIPOQ	Adiponectin, C1Q, And Collagen Domain Containing	0.0247	9
PPARA	Peroxisome Proliferator-Activated Receptor Alpha	0.0225	8
MAPK14	Mitogen-Activated Protein Kinase 14	0.0391	8
CCL5	C-C Motif Chemokine Ligand 5	0.0087	8
RELA	RELA Proto-Oncogene, NF-κB Subunit	0.0083	8
CYP19A1	Cytochrome P450 Family 19 Subfamily A Member 1	0.0166	6
ADA	Adenosine Deaminase	0.0178	5
GPR4	G Protein-Coupled Receptor 4	0.0070	2
LDLR	Low-Density Lipoprotein Receptor	0.0109	2
IL16	Interleukin 16	0.0272	2
SERPINF1	Serpin Family F Member 1	0.0625	2
TLR2	Toll-Like Receptor 2	0.0625	2
NINJ1	Ninjurin 1	0.0054	2
ACP5	Acid Phosphatase 5, Tartrate Resistant	0.1210	2
CCL3	C-C Motif Chemokine Ligand 3	0.0109	2
AGT	Angiotensinogen	0.0272	2

## Data Availability

The data that support the findings of this study are available on request from the corresponding author.

## Supplementary Materials

The following supporting information can be downloaded at: https://www.mdpi.com/article/10.3390/ijms26199374/s1.
