# Supplementary material for: Phytochemical Analysis and Phytometabolomic Profiling of Ficus lindsayana Leaf Extract with Evaluation of Antioxidant, Anti-Inflammatory, Cyto- and Genotoxic Activities
Source: Int J Mol Sci. 2025 Sep 25;26(19):9374. doi: 10.3390/ijms26199374 (PMC12524440; doi:10.3390/ijms26199374)
Supplement: Supplementary file 1 [file ijms-26-09374-s001.zip › ijms-3871345-SI.pdf]

International Journal of
Molecular Sciences

MDPI

Supplementary Data

Phytochemical Analysis and Phytometabolomic Profiling of *Ficus lindsayana* Leaf Extract with Evaluation of Antioxidant, Anti-Inflammatory, Cyto- and Genotoxic Activities

Arisa Imsumran [1,†], Woorawee Inthachat [2,†], Piya Temviriyanukul [2], Jirarat Karinchai [1], Tanongsak Laowanitwattana [1], Pensiri Buacheen [1], Ararat Jaiaree [1], Uthaiwan Suttisansanee [2], Ariyaphong Wongnoppavich [1] and Pornsiri Pitchakarn [1,*]

[1] Department of Biochemistry, Faculty of Medicine, Chiang Mai University, Muang Chiang Mai, Chiang Mai 50200, Thailand; arisa.bonness@cmu.ac.th (A.I.); jirarat.karin@gmail.com (J.K.); tanongsak.l@cmu.ac.th (T.L.); pensiri.bua@cmu.ac.th (P.B.); ararat.ja@gmail.com (A.J.); ariyaphong.w@cmu.ac.th (A.W.)
[2] Institute of Nutrition, Mahidol University, Salaya, Nakhon Pathom 73170, Thailand; woorawee.int@mahidol.ac.th (W.I.); piya.tem@mahidol.ac.th (P.T.); uthaiwan.sut@mahidol.ac.th (U.S.)
* Correspondence: pornsiri.p@cmu.ac.th; Tel.: +66-53-935325
† These authors contributed equally to this work.

 https://doi.org/10.3390/ijms26199374

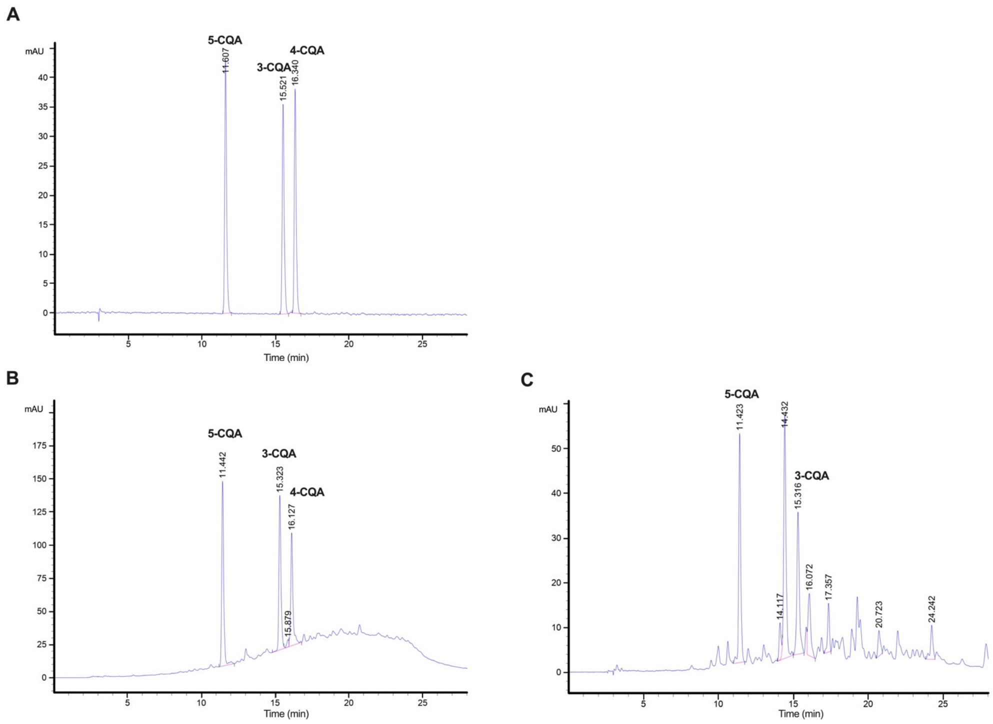

Figure S1. HPLC profiles of 3-,4-,5- CQA (**A**) FLA (**B**) and FLE (**C**).

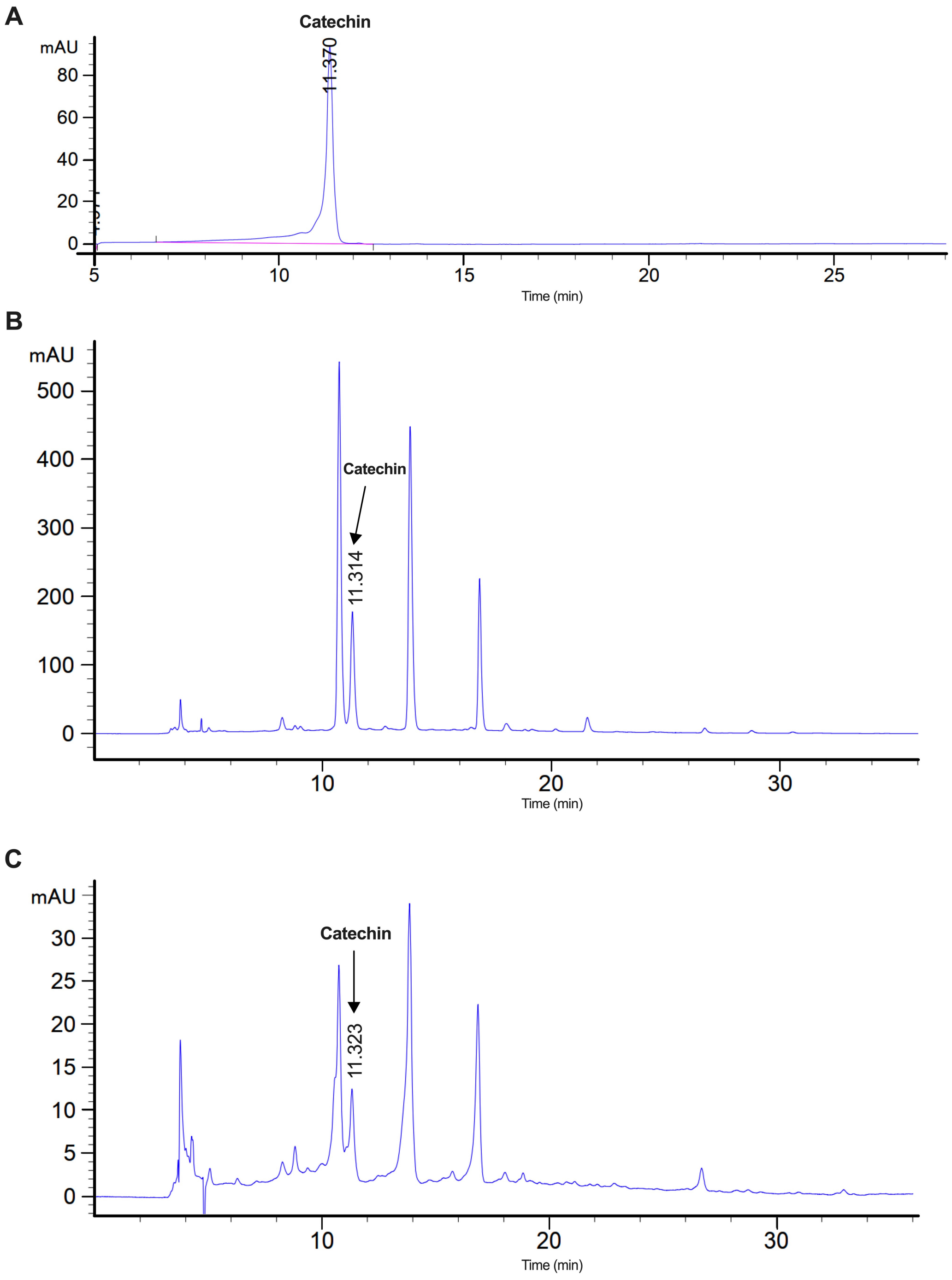

Figure S2. HPLC profiles of Catechin (**A**) FLA (**B**) and FLE (**C**).

Table S1. Specific primer sequences for RT-qPCR.

Gene	Forward primer (5'→3')	Reverse primer (5'→3')
COX2	TACTACACCAGGGCCCTTCC	CATATTTGAGCCTTGGGGGT
GAPDH	CTTTGTCAAGCTCATTTCCTGG	TCTTGCTCAGTGTCCTTGC
IL-1β	AAGGGCTGCTTCCCAACCTTTGAC	ATACTGCCTGCCTGAAGCTCTTGT
IL-6	ATCCAGTTGCCTTCTTGGGACTGA	TAAGCGTCCGACTTGTGAAGTGGT
iNOS	TCTTTGACGCTCGGAACTGTAGCA	ACCTGATGTTGCCATTGTTGGTGG
TNF-α	TCTCATGCACCACCATCAAGGACT	ACCACTCTCCCTTTGCAGAACTCA

Table S2. Information on bioactive constituents of FLA used in network pharmacology analysis.

Agents	Name	Lipinski Rules				Lipinski Violations ≤ 1	Bioavailability Score > 0.1
		MW <500 Da	H-bond acceptors <10	H-bond donors <5	MLOGP ≤ 4.15		
1	Erucamide	337.58	1	1	5.06	1	0.55
2	Chlorogenic acid	354.31	9	6	-1.05	1	0.11
3	1-Stearoyl-rac-glycerol	358.56	4	2	3.63	0	0.55
4	Trigonelline	137.14	2	0	0.33	0	0.55
5	Pantothenic acid	219.23	5	4	-0.8	0	0.56
6	Daidzein	254.24	4	2	1.08	0	0.55
7	2-Linoleoylglycerol	354.52	4	2	1.08	0	0.55
8	Stearamide	283.49	1	1	4.27	1	0.55
9	1-Palmitoyl-sn-glycero-3-phosphocholine	495.63	7	1	-1.13	0	0.55
10	Syringaldehyde	182.17	4	1	0.24	0	0.55
11	1H-Indole-4-carboxaldehyde	145.16	1	1	0.88	0	0.55
12	Nicotinic acid	123.11	3	1	-1.13	0	0.85

Table S3. Information on bioactive constituents of FLE used in network pharmacology analysis.

Agents	Name	Lipinski Rules				Lipinski Violations ≤ 1	Bioavailability Score > 0.1
		MW <500 Da	H-bond acceptors <10	H-bond donors <5	MLOGP ≤ 4.15		
1	1-Palmitoyl-sn-glycero-3-phosphocholine	496.64	8	3	3.64	2	0.17
2	9-Oxo-10E,12Z-octadecadienoic acid	278.43	3	1	4.35	0	0.55
3	Trigonelline	137.14	3	1	-0.92	0	0.55
4	Chlorogenic acid	354.31	7	5	-0.54	1	0.55
5	Pantothenic acid	219.23	5	3	-1.03	0	0.55
6	Adenine	135.13	5	5	-1.11	0	0.85
7	Monolinolenin	352.50	4	2	6.05	1	0.55
8	1H-Indole-4-carboxaldehyde	145.15	2	1	1.53	0	0.55
9	Friedelin	426.72	0	0	9.22	3	0.11
10	Campesterol	400.68	1	4	8.14	1	0.17
11	Indole	117.15	1	1	1.13	0	0.55
12	9,12-Octadecadiynoic acid	268.41	2	1	5.34	0	0.55
13	1-Monolinoleoyl-rac-glycerol	354.52	4	2	4.23	1	0.55
14	Stearamide	283.49	1	1	6.02	1	0.55
15	Benzyldodecyldimethylammonium cation	308.53	0	0	2.53	2	0.11
16	1-Stearoyl-rac-glycerol	256.54	4	2	6.33	1	0.55
17	Vitamin B6	169.18	3	3	-1.15	0	0.55
18	Rutin	664.56	16	10	-1.53	3	0.17
19	Naringenin	272.25	5	4	1.84	0	0.55
20	Kaempferol-3-O-rutinoside	594.52	12	10	-1.33	3	0.17
21	Vitamin B2	376.40	9	5	-1.97	2	0.17
22	1,5-Diazabicyclo4.3.0-non-5-ene	124.18	2	1	1.45	0	0.55
23	1H-Indole-3-carboxylic acid	161.16	3	2	1.25	0	0.55
24	Cinnamaldehyde	132.15	1	0	2.12	0	0.55
25	Pomiferin	420.52	6	5	3.84	1	0.55
26	7.alpha.,24(S)-Dihydroxycholesterol	414.65	3	2	7.52	1	0.17
27	2-Phenylbutyric acid	164.20	2	1	2.16	0	0.55
28	Isorhamnetin 3-neohesperidoside	624.55	15	10	-1.63	3	0.17
29	Butaprost	398.53	6	3	4.65	1	0.55
